# Unveiling the Electronic and Optoelectronic Behavior of Phenothiazine Derivatives through Theoretical Insights

**DOI:** 10.1021/acsomega.6c01934

**Published:** 2026-06-24

**Authors:** Murugesan Panneerselvam, Anantha Narayanan Sri Gayathri, Madhu Deepan Kumar, Iravatham Rama, Luciano T. Costa, Madhavan Jaccob

**Affiliations:** † MolMod-CS—Instituto de Química, 28110 Universidade Federal Fluminense, Campos Valonginho s/n, Centro, Niterói, Rio de Janeiro 24020-14, Brazil; ‡ Chemical Engineering Program (PEQ/COPPE), Federal University of Rio de Janeiro (UFRJ), Rio de Janeiro, Rio de Janeiro 21941-594, Brazil; § PG and Research Department of Chemistry, Seethalakshmi Ramaswami College, 366070 Affiliated to Bharathidasan University, Tiruchirappalli, Tamil Nadu 620 002, India; ∥ Department of Chemistry, K. Ramakrishnan College of Technology, Samayapuram, Tiruchirapalli, Tamil Nadu 621112, India; ⊥ Department of Chemistry & Computational Chemistry Laboratory, Loyola Institute of Frontier Energy (LIFE), 29843 Loyola College, Chennai, Tamil Nadu 600 034, India

## Abstract

A series of phenothiazine-based donor–π–acceptor derivatives (PTZ1–PTZ10) were systematically designed and investigated using density functional theory (DFT) and time-dependent DFT (TD-DFT) to elucidate structure–property relationships governing their optoelectronic performance. Strategic incorporation of aryl substituents with varying electronic character enables fine-tuning of molecular planarity, Frontier orbital energies and intramolecular charge transfer (ICT). Frontier molecular orbital analysis reveals efficient donor–acceptor separation across the series, with reduced band gaps and enhanced delocalization observed for PTZ6–PTZ10, promoting stronger ICT and improved light-harvesting capability. TD-DFT results indicate pronounced bathochromic shifts and high oscillator strengths for these derivatives, with absorption extending toward the visible region. Solvent-dependent studies further confirm strong solvatochromic behavior, highlighting the stabilization of charge-transfer excited states in polar environments. Excited-state analysis shows small singlet–triplet energy gaps (Δ*E*_ST_ = 0.155–0.417 eV), suggesting efficient intersystem crossing and exciton utilization. Charge-transport analysis demonstrates that PTZ7 and PTZ10 are favorable for hole transport, while PTZ2 and PTZ5 exhibit superior electron-transport properties, with several derivatives displaying balanced ambipolar characteristics. Notably, PTZ10 exhibits the highest light-harvesting efficiency, favorable injection driving force and strong charge separation, identifying it as the most promising candidate for photovoltaic applications. Complementary NCI-RDG and QTAIM analyses reveal that hydrogen bonding and dispersion interactions play key roles in stabilizing molecular conformations and facilitating electronic communication. Overall, this study provides a comprehensive framework for the rational design of phenothiazine-based chromophores, offering valuable insights for the development of efficient materials for dye-sensitized solar cells and related optoelectronic devices.

## Graphical abstract

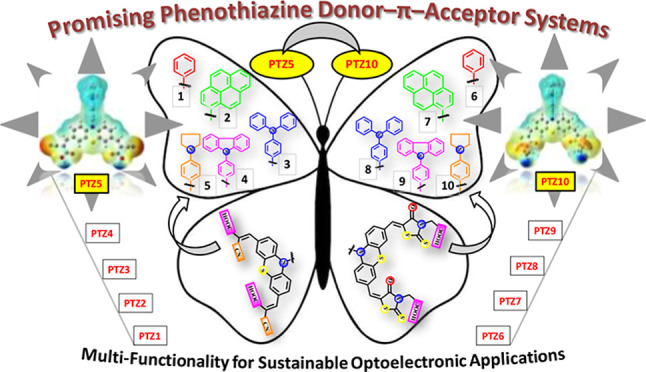

## 1 Introduction

Over the past decades, solar energy has emerged as a sustainable solution to environmental pollution and energy scarcity, driving the rapid development of various solar cell technologies.^1^ These technologies are generally categorized into three generations based on their materials and design principles. The first-generation solar cells are silicon-based devices, which are widely adopted in commercial and domestic applications due to their high and stable efficiencies.^2–4^ However, their widespread use is hindered by high production costs and complex purification processes. As a result, dye-sensitized solar cells (DSSCs), first introduced by Grätzel and O’Regan in 1991, have gained attention as a promising low-cost alternative.^5^ DSSCs, commonly referred to as Grätzel cells, have emerged as an up-and-coming class of photovoltaic devices, attracting considerable interest and growing attention.^6^ Within this category, dyes derived from the 10H-phenothiazine framework have gained notable prominence due to their facile structural tunability, economic viability and low environmental footprint.^7,8^ The incorporation of 10H-phenothiazine-based dyes into DSSCs was first introduced by Sun and co-workers in 2007.^9^ Organic dyes have become favorable alternatives to metal-complex sensitizers in DSSCs due to their high molar extinction coefficients, ease of molecular design, low synthesis cost and straightforward purification.^10,11^ Recent advancements have led to remarkable power conversion efficiencies (PCEs), with over 12% achieved by state-of-the-art organic dye-sensitized DSSCs.^12^ Notably, Sun’s group reported a PCE of 13.6% using single organic dyes,^13^ while Kakiage et al. achieved a record 14.3% in a cosensitized system.^14^

Despite these advancements, considerable efforts continue in optimizing the donor–π bridge–acceptor (D–π–A) framework. Molecular engineering strategies include tuning the donor, π-bridge, or acceptor units to enhance performance.^15,16^ Common donors include triphenylamine,^17^ indoline,^18^ carbazole,^19^ phenothiazine and phenoxazine,^20^ while π-bridges often involve thiophene derivatives, furan, pyrrole or fused-ring systems.^21^ Acceptors such as cyanoacetic acid, rhodanine-acetic acid and benzoic acid are widely employed.^22^ Additionally, modified architectures like D–D–π–A,^23^ D–A–π–A,^24^ D–π–D–A,^25^ A–π–D–π–A, and D–π–A_2_ have been explored to improve photovoltaic performance.^26^ In recent years, perovskite-based DSSCs have achieved higher PCEs than their inorganic,^27^ organic^28^ and natural dye counterparts.^29^ Despite this, the inherent limitations of perovskites, including poor stability and potential toxicity, have prompted increased attention toward natural dyes as promising alternatives.^30^ DSSCs hold significant potential for future energy solutions owing to their low environmental impact, compact design and utilization of cost-effective materials.^31^ However, the commercial scaling of DSSCs has progressed slowly, primarily due to their lower long-term stability and efficiency when compared to widely adopted silicon-based photovoltaics.^32^ DSSCs have emerged as a compelling class of photovoltaic (PV) devices, offering an attractive alternative to conventional technologies due to their low fabrication costs, relatively high PCEs and ease of production.^33^ Among the various components, the dye plays a critical role, directly impacting both the efficiency and long-term stability of the device.^34,35^ The ability to fine-tune the optical and electrochemical properties of organic dyes through targeted structural modifications has further accelerated research in this domain.^36^ A wide range of donor frameworks such as coumarin,^37^ indoline,^38^ hemicyanine,^39^ carbazole,^40^ triphenylamine,^41^ tetrahydroquinoline,^42^ porphyrin^43^ and phenothiazine^44^ have been explored for their application in DSSCs.^45^ Among these, PTZ has attracted particular interest owing to its unique structural features. It possesses a nonplanar heterotricyclic framework containing electron-rich sulfur and nitrogen atoms, rendering it a highly effective electron donor.^46,47^ This nonplanarity also minimizes dye aggregation on the semiconductor surface, further improving device performance.^48^ Moreover, phenothiazine offers synthetic flexibility for the introduction of substituents at multiple positions, allowing precise control over electronic properties.^49,50^

This study aims to elucidate the structure–property relationships of phenothiazine-based donor–π–acceptor derivatives through systematic π-aryl substitution and to investigate their electronic and optoelectronic properties using DFT, TD-DFT, QTAIM, and NCI analyses. Key objectives include evaluating Frontier orbitals, band gaps, charge separation, and reorganization energies to identify efficient light-harvesting, charge-transfer, and charge-transport candidates, as well as correlating molecular polarity and intermolecular interactions with performance. These insights will guide the rational design of phenothiazine-based chromophores with tunable electronic responses, stability, and multifunctionality for advanced optoelectronic applications.

## 2 Computational Methodology

DFT has proven to be a robust and computationally efficient methodology for evaluating the total energies and exploring the electronic structures of molecular systems.^51–53^ Geometry optimizations were performed using the long-range corrected CAM-B3LYP functional,^54^ combined with the def2-TZVP basis set. Previous studies^55–59^ have shown that CAM-B3LYP yields improved accuracy in predicting absorption energies and electronic transitions with good agreement with experimental results. Therefore, this level of theory is considered appropriate for investigating the optoelectronic properties of the designed systems.

All quantum chemical calculations were performed using the ORCA software suite.^60^ TD-DFT computations were subsequently conducted with the same functional and basis set to simulate the electronic absorption spectra.^61,62^ Noncovalent interaction (NCI) analyses were performed using the Multiwfn 3.7 program,^63,64^ while the Quantum Theory of Atoms in Molecules (QTAIM) analysis was executed via the AIM2000 software package.^65–67^ To further elucidate the electronic characteristics and assess the optoelectronic suitability of the studied compounds for optoelctronic applications,^68,69^ The light-harvesting efficiency (LHE), an important parameter for evaluating the performance of organic chromophores in dye-sensitized solar cells (DSSCs), was estimated from the oscillator strength (*f*) using the relation

1 $${{LHE=1-10}^{-}}^{f}$$

LHE reflects the ability of the molecule to absorb incident photons and promote electron injection into the conduction band of the semiconductor. The ionization potential (IP_v_) and electron affinity (EA_v_) were calculated using both vertical and adiabatic approaches based on total energy differences between neutral, cationic and anionic species. These parameters provide important insights into the charge injection and charge transport characteristics of the molecules.

2 $$IP_{a}=E_{+}^{+}-E_{0}^{0}$$

3 $$IP_{v}=E_{+}^{0}-E_{0}^{0}$$

4 $$EA_{a}=E_{0}^{0}-E_{-}^{-}$$

5 $$EA_{v}=E_{0}^{0}-E_{-}^{0}$$

The charge transport properties were further evaluated by calculating the internal reorganization energies for hole (λ_hole_) and electron (λ_electron_) transport within the framework of Marcus theory. The reorganization energies were computed using the following expressions

6 $$\lambda_{hole}=\left[E^{+}\left(M_{0}\right)-E^{+}\left(M^{+}\right)\right]+\left[E\left(M^{+}\right)-E\left(M_{0}\right)\right]$$

7 $$\lambda_{electron}=\left[E^{-}\left(M_{0}\right)-E^{-}\left(M^{-}\right)\right]+\left[E\left(M^{-}\right)-E\left(M_{0}\right)\right]$$

where *E*, *E* ^*+*^ and *E* ^*–*^ represent the total energies of the neutral, cationic, and anionic species, respectively. *M*_0_, *M* ^+^ and *M* ^–^ denote their optimized geometries, while *E* ^+^ (*M*_0_) and *E* ^–^ (*M*_0_) correspond to the energies of the charged species evaluated at the optimized neutral geometry. Lower values of λ_hole_ and λ_electron_ indicate more efficient hole and electron transport, respectively.^70^ In addition, the excited-state lifetime (τ in ns) was estimated from the oscillator strength and transition energy using the radiative decay rate relationship based on the Strickler–Berg approach.^71^

## 3 Results and Discussion

The design of the phenothiazine derivatives (PTZ1–PTZ10) is based on a rational π-conjugation extension strategy, where the phenothiazine core is functionalized with various aryl substituents to modulate its electronic properties. Phenothiazine is a well-known electron-rich donor moiety with excellent charge transport characteristics; however, its optoelectronic performance can be further enhanced through structural modification.^72–76^ In this work, different π-aryl groups bearing electron-donating and electron-withdrawing substituents are introduced to systematically tune the HOMO–LUMO energy levels, intramolecular charge transfer (ICT), and optical absorption behavior. The incorporation of extended aromatic systems promotes effective π-electron delocalization across the molecular framework, thereby improving light-harvesting ability and charge transfer efficiency.

Scheme 1 illustrates the structural diversity of phenothiazine (PTZ)-based donor architectures (PTZ1–PTZ10), strategically designed for advanced optoelectronic applications. Each molecular system is constructed around a central phenothiazine core, well-known for its strong electron-donating ability and conformational flexibility. In these structures, different aryl substituents are introduced at the top position of the PTZ framework to fine-tune the electronic and photophysical properties.

**Scheme 1:**
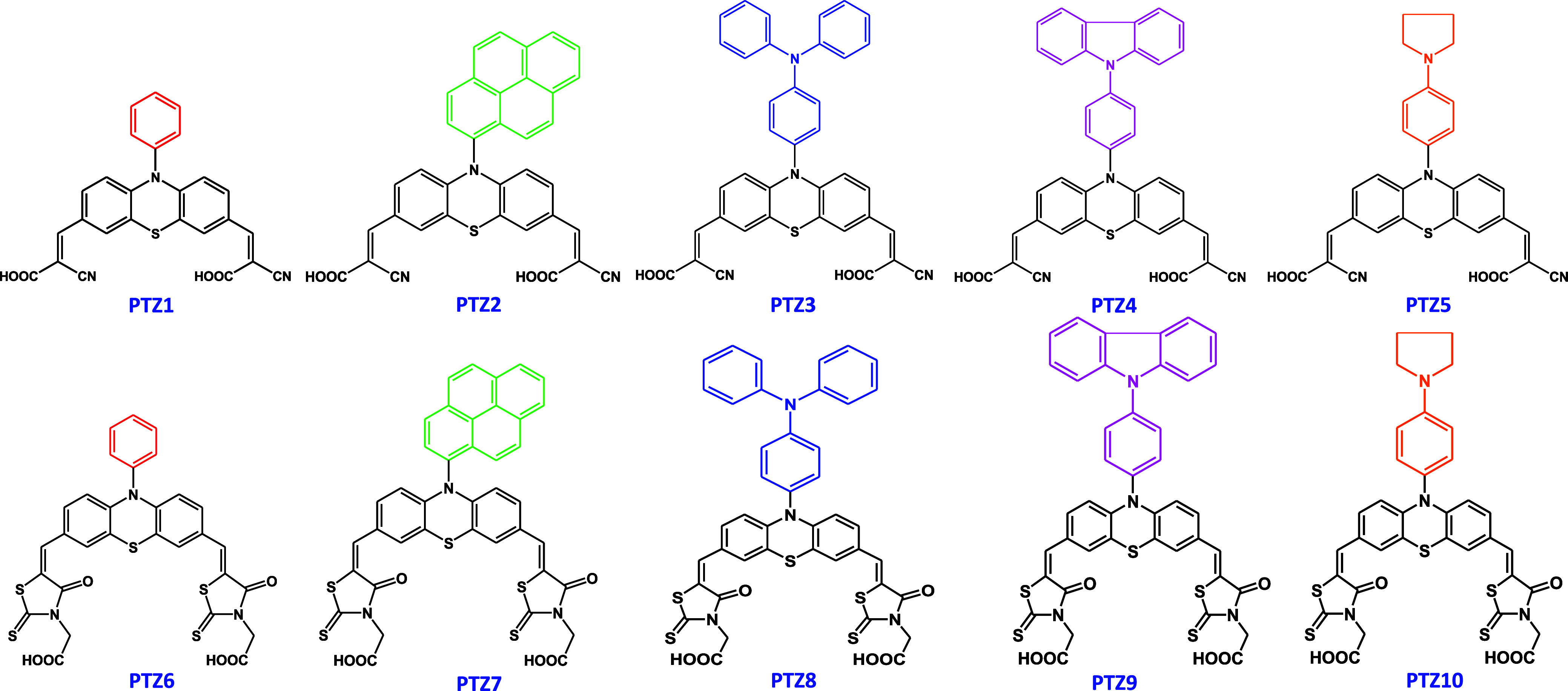
Schematic Representation of PTZ-Based Donor Molecules (PTZ1–PTZ10) Functionalized with Various π-conjugated Aryl Units

### 3.1 Optimized Geometries: Structural Parameters and Hydrogen-Bonding Interactions

The optimized geometries of PTZ1–PTZ10, along with their selected bond lengths, bond angles, and hydrogen-bonding interactions, are presented in Figure 1. PTZ1 exhibits a nearly planar geometry with a small torsional angle (8.12°). The phenothiazine core retains bond lengths within typical aromatic ranges (*d*_1_ = 1.436 Å, *d*_2_ = *d*_3_ = 1.401 Å), while hydrogen-bond donor–acceptor distances of 2.001–2.003 Å and 2.263–2.264 Å indicate moderate stabilization through O···H interactions. PTZ2 adopts a slightly twisted conformation (θ = −10.49°), accompanied by marginal bond shortening (*d*_2_ = 1.399 Å). Its hydrogen-bonding profile includes short contacts of 2.001–2.005 Å and 2.262–2.264 Å, along with extended interactions near 2.790 Å, favoring intermolecular packing. PTZ3 displays greater torsional distortion (θ = −13.24°), with bond metrics close to PTZ1 but a reduced *d*_2_ (1.399 Å), suggesting localized conjugation. PTZ4 features a nonplanar structure (θ = −12.93°), stabilized by electron-rich substituents. Bond distances (*d*_1_ = 1.434 Å, *d*_2_ = 1.402 Å) remain consistent, while hydrogen-bond lengths span 2.005–2.265 Å, enabling directional intermolecular interactions. PTZ5 maintains partial planarity (θ = 9.57°), with shorter *d*_2_ and *d*_3_ values (1.398 Å). Its hydrogen-bonding interactions range from 2.006–2.297 Å, with additional long-range contacts extending to 2.623 Å, suggesting enhanced stability through donor–acceptor complementarity. PTZ6 shows pronounced torsional distortion (θ = 13.42°), with shortened bond distance (1.401 Å). Its hydrogen-bonding network spans 2.006–2.388 Å, including bifurcated interactions up to 2.618 Å. PTZ7 adopts a gently tilted geometry (θ = −8.81°), with minimal bond variation (*d*_3_ = 1.401 Å). Hydrogen-bond distances of 2.027–2.390 Å and 2.618–2.630 Å reflect a balanced distribution of donor and acceptor functionalities, contributing to structural stability. PTZ8 retains near planarity (θ = 8.79°), with bond parameters consistent with PTZ1 (*d*_2_ = *d*_3_ = 1.401 Å). Its hydrogen-bonding network comprises both short contacts (2.033–2.040 Å) and longer interactions (∼2.634 Å), suggesting robust intermolecular stabilization. PTZ9 exhibits a highly twisted conformation (θ = −15.87°), with localized electron delocalization evidenced by shortened bond distances (1.401 Å). Hydrogen-bond donor–acceptor distances of 2.015–2.383 Å and ∼2.627 Å indicate a mixed network of strong and weak interactions. PTZ10 shows the greatest torsional deviation (θ = −14.43°), likely due to bulky or polar substituents. While the core bond lengths remain stable (1.402 Å), its hydrogen-bonding interactions range from 2.033–2.057 Å and extend to 2.628 Å, reflecting the coexistence of short- and long-range interactions relevant to solid-state assembly. Overall, the structural and hydrogen-bonding analyses across PTZ1 to PTZ10 demonstrate that substituent variation systematically modulates planarity, bond parameters, thereby influencing molecular stability and optoelectronic performance.

**Figure 1:**
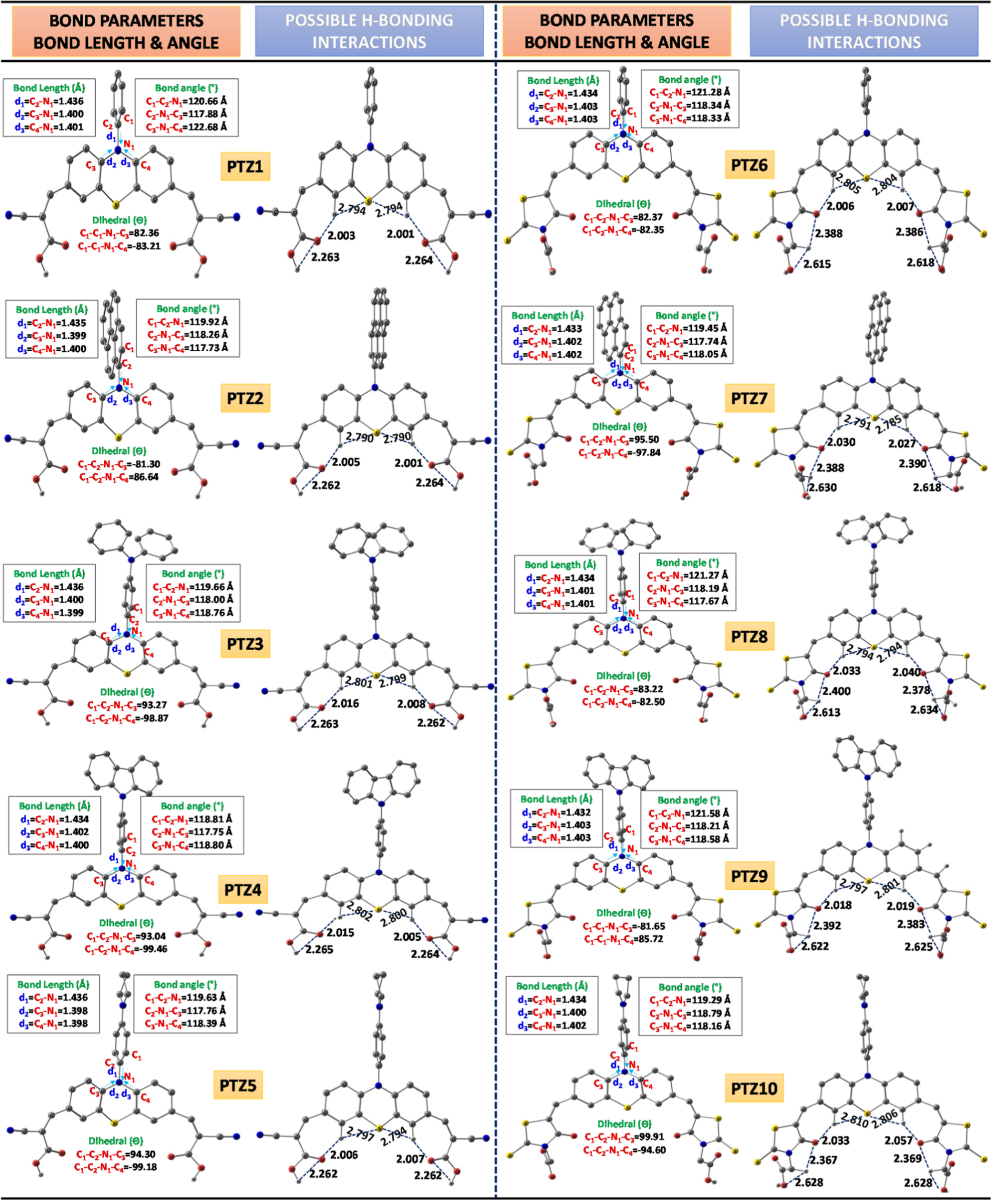
Optimized geometries of the PTZ molecules along with their bond parameters (bond lengths (Å), bond angles (°) and dihedral angles (°)) and respective hydrogen bonding interactions.

### 3.2 FMOs and *E*_g_ Analysis: Donor–Acceptor Separation and ICT Characteristics

Frontier molecular orbital (FMO) analysis of PTZ1–PTZ10 provides detailed insight into their electronic structures and intramolecular charge-transfer (ICT) characteristics (see Figure 2). For PTZ1, the HOMO – 2 and HOMO – 1 are localized on the phenothiazine (PTZ) donor, while the HOMO extends partially onto the π-bridge; in contrast, the LUMO, LUMO + 1, and LUMO + 2 are predominantly distributed over the acceptor unit, indicating effective donor–acceptor separation.

**Figure 2:**
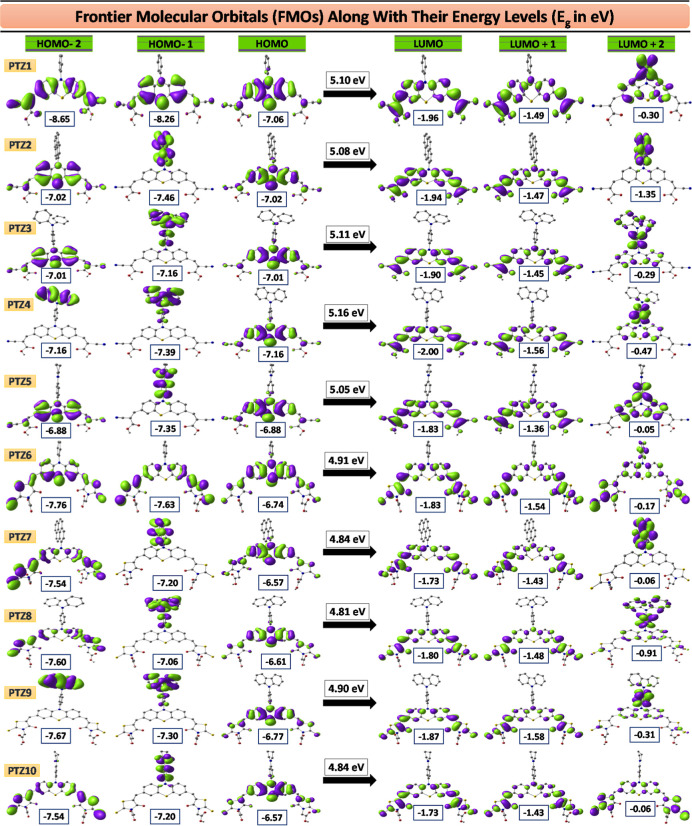
Frontier molecular orbitals (FMOs) from HOMO – 2 to LUMO + 2 for PTZ molecules along their energy gap (*E*_g_ in eV) values.

This general orbital distribution is maintained across the series, supporting efficient charge separation. The computed Frontier orbital energy gaps (*E*_g_) at the B3LYP level (See the Supporting Information, Table S1) show HOMO energies in the range −6.43 to −7.08 eV and LUMO energies from −1.97 to −2.24 eV (vs vacuum). These values are appropriately aligned with the *I* ^–^/*I*_3_^–^ redox potential (−4.60 eV) and the TiO_2_ conduction band edge (−4.00 eV), ensuring thermodynamically favorable dye regeneration and electron injection, respectively, and confirming the suitability of all dyes for DSSC applications. Although range-separated functionals with optimal tuning can provide more accurate absolute orbital energies, CAM-B3LYP is sufficient here for capturing consistent relative trends across the series.^77–79^ Across PTZ1–PTZ5, the FMOs remain relatively localized, giving HOMO–LUMO gaps in the range of 5.05–5.16 eV, with PTZ4 exhibiting the largest gap due to reduced conjugation. PTZ2 and PTZ3 show similar orbital distributions with slight extension of the HOMO over the bridge, resulting in comparable gaps (5.08 and 5.11 eV, respectively) and modestly improved ICT. In PTZ5, partial HOMO–LUMO overlap is observed, with a gap of 5.05 eV. In contrast, PTZ6–PTZ10 display increased delocalization across the donor–π–acceptor framework, leading to narrower gaps (4.81–4.91 eV) and enhanced ICT. Notably, PTZ8 exhibits the smallest gap (4.81 eV) with the most extended orbital delocalization, while PTZ7 and PTZ10 (4.84 eV) also show significant donor–acceptor communication. PTZ9 maintains a balanced delocalization with a gap of 4.90 eV. These trends are governed by substituent effects: electron-donating groups raise HOMO energies and reduce the band gap, enhancing ICT and promoting bathochromic shifts, whereas electron-withdrawing groups stabilize the Frontier orbitals and increase the gap. It is important to note that the HOMO–LUMO gaps obtained using CAM-B3LYP (4.81–5.16 eV) correspond to ground-state Kohn–Sham orbital gaps, which typically overestimate optical gaps and do not directly represent photoactive excitations. In DSSCs, light absorption is determined by TD-DFT vertical excitation energies. Consistently, TD-DFT results show strong visible-region absorption arising from ICT transitions (donor → acceptor), with significant oscillator strengths and pronounced bathochromic shifts, particularly for PTZ6–PTZ10, confirming efficient light harvesting. Therefore, HOMO–LUMO gaps are used here to rationalize electronic trends, while optical and device-relevant properties are discussed based on TD-DFT results in the next section.

### 3.3 Electronic Spectral Analysis: Structural Effects on Absorption, Emission, and Light-Harvesting Performance

The electronic spectra of the PTZ1–PTZ10 series reveal substantial variations in both the absorption and emission (λ_max/emi_) spectra and oscillator strengths (*f*_0_), reflecting the influence of structural modifications on their electronic transitions (see Tables 1 and 2). The first absorption band (λ_1_) is observed between 405.6 and 432.5 nm. Among these, PTZ8 exhibits the most red-shifted λ_1_ at 432.5 nm with a high *f*_0_ of 0.867, indicating enhanced π-conjugation and stronger intramolecular charge transfer (ICT) character. In contrast, PTZ4 shows the most blue-shifted λ_1_ at 405.6 nm with a relatively lower *f*_0_ of 0.504, suggesting a less efficient ICT. Notably, the λ_1_ values for PTZ6 to PTZ10 are consistently red-shifted compared to PTZ1 to PTZ5 and all of them exhibit significantly higher *f*_0,_ ranging from 0.85–0.91, signifying stronger light absorption and more efficient electronic transitions. These features point to an enhanced donor–acceptor interaction and extended delocalization in these molecules. The secondary absorption bands (λ_2_–λ_4_), located between 330.4 and 380.8 nm, display a contrasting trend. In PTZ6 to PTZ10, these transitions are associated with very low *f*_0_ (0.0001–0.0004), indicating symmetry-forbidden or weakly allowed transitions, potentially involving local excitation within either donor or acceptor regions. On the other hand, PTZ1–PTZ5 exhibit moderately allowed transitions in these bands with relatively higher *f*_0_ values, for PTZ2 (λ_2_ = 335.1 nm, *f*_0_ = 0.435), suggesting better orbital overlap. Additionally, PTZ1 and PTZ2 show particularly strong λ_4_ bands (for PTZ1, λ_4_ = 290.3 nm, *f*_0_ = 1.026), in contrast to PTZ3, which shows a significantly weaker transition (λ_4_ = 305.0 nm, *f*_0_ = 0.12). The UV–vis spectral analysis of PTZ1 to PTZ10 highlights the critical role of structural modification in tuning their optical and electronic properties. The consistent red shift and higher *f*_0_ observed in PTZ6 to PTZ10 suggest enhanced π-conjugation and stronger intramolecular charge transfer (ICT), attributed to improved donor–acceptor interactions and extended electronic delocalization. In contrast, PTZ1 to PTZ5 exhibit comparatively weaker ICT features with moderate absorption intensities. The significantly low *f*_0_ for higher–energy transitions in PTZ6 to PTZ10 further confirms the presence of symmetry-restricted excitations, while PTZ1 to PTZ5 show better orbital overlap and local excitation behavior. This variation can also be correlated with the number of carboxylic acid groups, where compounds containing a single carboxylic acid group exhibit enhanced π-conjugation and intramolecular charge transfer, resulting in a bathochromic shift toward the visible region, whereas the presence of two carboxylic acid groups reduces effective conjugation, leading to absorption predominantly in the UV region. The fluorescence emission spectra of PTZ1 to PTZ10 exhibit distinct shifts in emission maxima (λ_max_) and variations in *f*_0_, influenced by the electronic structure and substituents present on the phenothiazine core. The λ_1_ ranges from 521.0 nm for PTZ4 to 537.7 nm for PTZ10. Among these, PTZ10 shows the most red-shifted emission at 537.7 nm with the highest *f*_0_ = 0.600, indicating an efficient relaxation process likely due to extended π-conjugation and stronger donor–acceptor (D–A) interactions. Similarly, PTZ6–PTZ10 exhibit red-shifted λ_1_ values ranging from 529.8 to 537.7 nm and significantly higher *f*_0_ values, which range from 0.562 to 0.600 nm, compared to PTZ1 to PTZ5, ranging from 0.337 to 0.360 nm, reflecting enhanced electronic delocalization and fluorescence quantum efficiency. Secondary emission bands (λ_2_ and λ_3_) appear in the ranges of ∼372–400 nm and ∼328–384 nm, respectively. For PTZ1–PTZ5, λ_2_ and λ_3_ transitions have moderate oscillator strengths (*f*_0_ = 0.179–0.290 and *f*_0_ = 0.016–0.101), indicating active excited-state emissions likely associated with locally excited (LE) states.

**Table 1:** Calculated Electronic Properties of PTZ Derivatives, Including Absorption Wavelengths (λ_1_–λ_4_, nm) and Corresponding Oscillator Strengths (*f*_0_)­^a^

molecules	λ_1_	*f*_0_	λ_2_	*f*_0_	λ_3_	*f*_0_	λ_4_	*f*_0_
PTZ1	413.2	0.503	331.2	0.224	308.4	0.316	290.3	1.026
PTZ2	414.7	0.518	335.1	0.435	297.8	0.496	290.6	1.024
PTZ3	410.3	0.531	333.4	0.375	308.6	0.282	305.0	0.120
PTZ4	405.6	0.504	330.4	0.313	306.8	0.310	289.4	0.958
PTZ5	418.0	0.547	334.6	0.287	310.1	0.344	290.6	0.965
PTZ6	419.6	0.869	380.3	0.0001	380.2	0.0001	349.2	0.769
PTZ7	431.0	0.853	379.9	0.0001	379.8	0.0002	356.8	0.948
PTZ8	432.5	0.867	379.6	0.0002	379.4	0.0002	356.2	0.870
PTZ9	420.7	0.860	380.8	0.0002	380.7	0.0001	351.0	0.878
PTZ10	426.8	0.907	379.2	0.0004	378.2	0.0004	354.2	0.824

a Here, λ_1_–λ_4_ denote the first four electronic transitions, where λ_1_ represents the lowest-energy (longest-wavelength) excitation governing the absorption onset, while λ_2_–λ_4_ correspond to higher-energy transitions.

**Table 2:** Calculated Electronic Properties of PTZ Derivatives, Including Emission Wavelengths (λ_1_–λ_4_, nm) and Corresponding Oscillator Strengths (*f*_0_)­^a^

molecules	λ_1_	*f*_0_	λ_2_	*f*_0_	λ_3_	*f*_0_	λ_4_	*f*_0_
PTZ1	522.3	0.337	372.5	0.179	332.0	0.026	310.5	1.784
PTZ2	521.6	0.339	375.1	0.290	328.8	0.101	310.7	0.089
PTZ3	525.8	0.345	375.4	0.262	332.5	0.037	310.9	1.723
PTZ4	521.0	0.324	372.7	0.238	332.5	0.016	310.8	1.709
PTZ5	528.0	0.360	376.0	0.223	332.8	0.049	311.1	1.736
PTZ6	529.8	0.576	393.9	0.410	383.6	0.0003	355.3	1.089
PTZ7	530.0	0.572	397.2	0.571	382.9	0.0002	377.1	0.018
PTZ8	532.6	0.599	396.9	0.529	382.7	0.0002	355.0	1.080
PTZ9	527.6	0.562	393.7	0.506	384.0	0.0003	355.1	1.091
PTZ10	537.7	0.600	399.2	0.469	381.9	0.0004	356.1	1.059

a Here, λ_1_–λ_4_ denote the first four electronic transitions, where λ_1_ represents the lowest-energy (longest-wavelength) excitation governing the absorption onset, while λ_2_–λ_4_ correspond to higher-energy transitions.

In contrast, PTZ6 to PTZ10 show a clear bathochromic shift in λ_2_ (399.2 nm for PTZ10) and a dramatic drop in *f*_0_ for λ_3,_ ranging from 0.0002 to 0.0004, suggesting forbidden or symmetry-restricted transitions, typical of charge transfer (CT) states where nonradiative processes dominate. A notable emission feature is the intense λ_4_ band (310 to 356 nm) seen in most PTZ derivatives, particularly PTZ1 (λ_4_ = 310.5 nm, *f*_0_ = 1.784), PTZ3 (310.9 nm, *f*_0_ = 1.723), and PTZ5 (311.1 nm, *f*_0_ = 1.736). These strong emissions indicate higher–energy transitions likely linked to π → π* or intraligand fluorescence. Although the majority of the compounds exhibit absorption predominantly in the UV region, selected derivatives (PTZ6–PTZ10) show noticeable bathochromic shifts toward the visible region along with enhanced oscillator strengths, indicating improved light-harvesting capability. However, further structural modification is required to achieve optimal absorption characteristics for efficient solar cell applications.

### 3.4 Solvent-Dependent Absorption and Emission Properties: Solvatochromic Effects

The absorption and emission properties of PTZ1–PTZ10 display pronounced solvatochromic behavior, demonstrating the significant impact of solvent polarity on their photophysical characteristics, as illustrated in Figure 3 and Tables S2–S12 (see the Supporting Information). In general, both absorption and emission maxima undergo a systematic red-shift (bathochromic shift) with increasing solvent polarity, following the order: toluene (ε = 2.4) < chloroform (ε = 4.8) < dichloromethane (ε = 9.1) < methanol (ε = 33) ≈ acetonitrile (ε = 36). This trend indicates that the excited states of PTZ derivatives are more stabilized in polar solvents than in nonpolar environments, consistent with a pronounced intramolecular charge transfer (ICT) character. For absorption spectra, the λ_abs_ values increase progressively from nonpolar to polar solvents for all derivatives. For instance, PTZ1 shows a shift from 426.6 nm in toluene to 440.6 nm in acetonitrile, while PTZ8 exhibits a more pronounced shift from 448.7 to 464.4 nm. Among the series, PTZ7–PTZ10 display the most red-shifted absorption maxima (up to 464.4 nm), reflecting enhanced conjugation and stronger donor–acceptor interactions. In contrast, PTZ4 consistently shows the most blue-shifted absorption (418.5–432.3 nm), suggesting relatively weaker electronic delocalization. A similar solvent-dependent trend is observed in emission spectra, with λ_em_ values also shifting to longer wavelengths in more polar solvents. For example, PTZ10 exhibits emission maxima ranging from 560.6 nm (toluene) to 583.7 nm (acetonitrile), indicating significant stabilization of the excited state. The magnitude of emission shifts is generally larger than that of absorption, leading to increased Stokes shifts in polar solvents. This behavior further supports the presence of a highly polarized excited state and efficient ICT process. Notably, PTZ6–PTZ10 exhibit significantly red-shifted emission (up to 583.7 nm) compared to PTZ1–PTZ5, indicating improved charge separation and stronger excited-state relaxation. Among all derivatives, PTZ10 shows the longest emission wavelength across all solvents, suggesting the most stabilized excited state and strongest ICT character.

**Figure 3:**
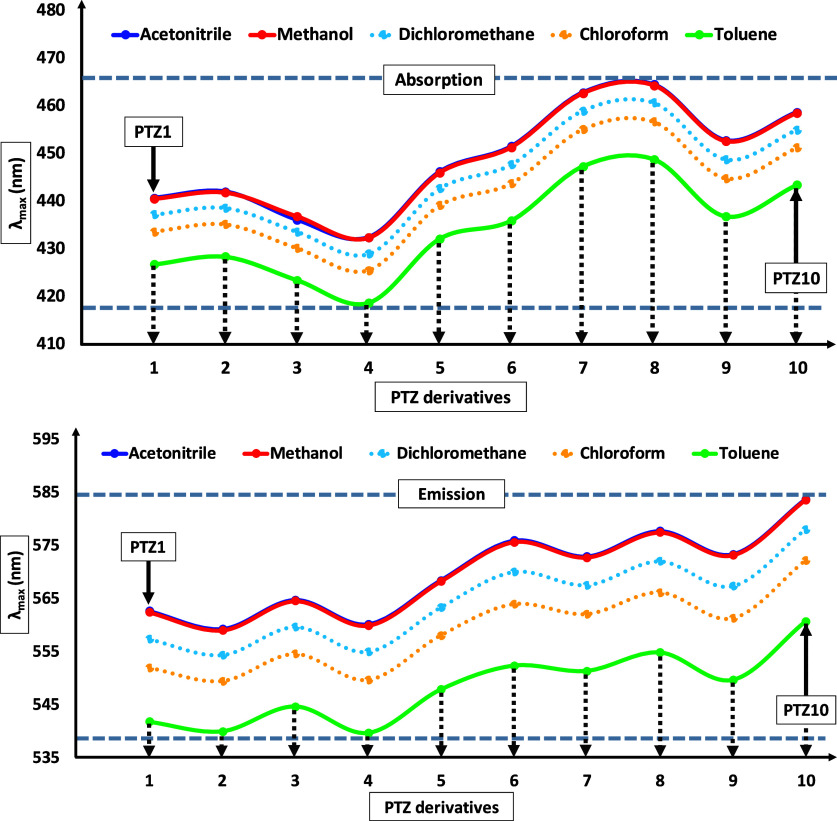
Calculated absorption (λ_abs_) and emission (λ_em_) wavelengths (nm) of PTZ derivatives in different solvents with varying dielectric constants (ε). Acetonitrile (ε = 36) and methanol (ε = 33) represent polar solvents, dichloromethane (ε = 9.1) and chloroform (ε = 4.8) are moderately polar, while toluene (ε = 2.4) is nonpolar. The reported values correspond to the lowest-energy electronic transitions for absorption and the corresponding emission maxima.

Conversely, PTZ2 and PTZ4 display relatively shorter emission wavelengths, consistent with weaker donor–acceptor interactions. Overall, the observed solvatochromic trends confirm that the optical properties of PTZ derivatives are highly sensitive to solvent polarity, with polar media enhancing both absorption and emission through stabilization of charge-transfer states. The larger shifts in emission relative to absorption highlight significant excited-state reorganization, making PTZ7–PTZ10 particularly promising candidates for optoelectronic and fluorescence-based applications.

### 3.5 Singlet–Triplet Excited-State Energetics and Δ*E*_ST_ Analysis

The singlet and triplet excitation energies obtained from the PBE functional provide useful insight into the optoelectronic properties of the PTZ derivatives (See Table S13 (Supporting Information)). The singlet excited state (S_1_) energies lie in the range of 1.705–1.951 eV, placing all the molecules within the visible region, which is desirable for light absorption and emission applications. The corresponding triplet state (T_1_) energies range from 1.375 to 1.579 eV, indicating that these systems can effectively access triplet states, an important factor for efficient exciton utilization in optoelectronic devices. An important parameter influencing device performance is the singlet–triplet energy gap (Δ*E*_ST_), which varies from 0.155 to 0.417 eV across the series. Among the studied molecules, PTZ3 shows the lowest Δ*E*_ST_ value (0.155 eV), suggesting that it can facilitate efficient intersystem crossing (ISC). On the other hand, PTZ5 (0.332 eV) and PTZ10 (0.385 eV) exhibit moderate energy gaps, indicating a good balance between exciton stability, which is beneficial for stable device performance. The relatively small Δ*E*_ST_ values observed for these compounds suggest that most of the PTZ derivatives are capable of efficient exciton conversion, which can contribute to improved electroluminescence efficiency in practical optoelectronic applications.

### 3.6 Global Reactivity Descriptors and Electronic Properties

To rationalize the electronic behavior and optoelectronic suitability of the designed phenothiazine-based donor–π–acceptor systems (PTZ1–PTZ10), the HOMO, LUMO, *E*_g_, global hardness (η), softness (*S*), chemical potential (μ), electrophilicity index (ω) and dipole moment are summarized in Table 3. The HOMO energy levels for the PTZ series range from −7.16 eV (PTZ4) to −6.57 eV (PTZ7 and PTZ10), while the corresponding LUMO energies span from −2.00 eV (PTZ4) to −1.73 eV (PTZ7 and PTZ10), indicating a systematic modulation of the Frontier orbitals with structural variation. The *E*_g_ falls within the range of 4.81–5.16 eV. PTZ8 exhibits the smallest *E*_g_ (4.81 eV), suggesting enhanced electron delocalization and a greater propensity for intramolecular charge transfer (ICT), which is desirable for efficient photoinduced charge separation in dye-sensitized systems. The η ranges narrowly from 2.41 to 2.58 eV across the series, indicating similar reactivity trends. The softness values consistently around 0.20 eV, indicate that these molecules are moderately polarizable with PTZ7, PTZ8 and PTZ10 showing slightly higher softness values (−0.21 eV), aligning with their lower band gaps and enhanced donor capacity. This increased softness may contribute to improved electronic coupling with acceptor units and semiconductor surfaces. The chemical potential (μ) shows a less negative value from PTZ4 (−4.58 eV) to PTZ10 (−4.15 eV), implying a transition from strong electron-withdrawing to more electron-rich systems. Notably, the less negative μ values observed for PTZ7, PTZ8 and PTZ10 suggest enhanced electron-donating abilities, thereby favoring effective electron injection into the conduction band of semiconductors such as TiO_2_. The electrophilicity index (ω) is highest for PTZ4 (4.06 eV) and lowest for PTZ10 and PTZ7 (3.55 eV). This trend suggests that PTZ4 behaves as the strongest electrophile within the series, while PTZ7 and PTZ10 are the most nucleophilic. Lower ω values are advantageous in photovoltaic applications, as they indicate lower tendencies for recombination and higher stability of the injected charge. The calculated dipole moments range from 5.25 D (PTZ9) to 10.48 D (PTZ5), with higher values indicating better solubility, stronger intermolecular interactions and improved alignment at semiconductor–dye interfaces. PTZ3, PTZ5 and PTZ10, with dipole moments exceeding 8.0 D, are expected to exhibit better anchoring and ordering on metal oxide surfaces, thereby contributing to enhanced dye loading and reduced charge recombination.

**Table 3:** Computed Quantum Chemical Descriptors of (*E*_g_), Softness (*S*), Hardness (η), Chemical Potential (μ) and Electrophilicity Index (ω) Along with the Energy Gap (*E*_g_) and Dipole Moment (*D*) for PTZ Molecules

molecules	HOMO	LUMO	*E*_g_	*S*	η	μ	ω	dipole
PTZ1	–7.06	–1.96	5.10	0.20	2.55	–4.51	3.98	6.62
PTZ2	–7.02	–1.94	5.08	0.20	2.54	–4.48	3.95	7.50
PTZ3	–7.01	–1.90	5.11	0.20	2.56	–4.46	3.88	8.41
PTZ4	–7.16	–2.00	5.16	0.19	2.58	–4.58	4.06	5.59
PTZ5	–6.88	–1.83	5.05	0.20	2.53	–4.36	3.75	10.48
PTZ6	–6.74	–1.83	4.91	0.20	2.46	–4.29	3.74	6.62
PTZ7	–6.57	–1.73	4.84	0.21	2.42	–4.15	3.55	7.09
PTZ8	–6.61	–1.80	4.81	0.21	2.41	–4.21	3.67	8.21
PTZ9	–6.77	–1.87	4.90	0.20	2.45	–4.32	3.80	5.25
PTZ10	–6.57	–1.73	4.84	0.21	2.42	–4.15	3.55	10.39

Collectively, the combined electronic and reactivity descriptors suggest that PTZ7, PTZ8 and PTZ10 exhibit a superior balance of small band gap, moderate hardness, high chemical softness, favorable chemical potential and elevated dipole moments.

### 3.7 NCI–RDG and QTAIM Analysis: Weak Interactions, Stability and Structural Organization

The NCI (noncovalent interactions) analysis, performed through reduced density gradient (RDG) isosurface mapping in conjunction with sign (λ_2_)­ρ scatter plots for the PTZ1–PTZ10 series, provides a detailed visualization of the weak interaction landscape and its role in governing molecular conformation, stability and packing behavior (see Figures 4, S1 and S2 and the Supporting Information Table S14). The color-coded isosurfaces distinctly differentiate the interaction regimes: intense blue regions (negative sign­(λ_2_)­ρ) correspond to strong attractive forces like classical hydrogen bonding and dipole–dipole interactions, predominantly localized around heteroatom centers whereas extended green zones (sign­(λ_2_)­ρ ≈ 0) signify dispersion-dominated van der Waals interactions, which are particularly pronounced between the PTZ core and its aryl substituents, suggesting efficient π–π stacking contributions. In contrast, red regions (positive sign­(λ_2_)­ρ) mark steric congestion, especially at ortho-substituted sites, potentially influencing torsional flexibility and electronic coupling between donor and acceptor fragments. The corresponding scatter plots consistently display three distinct regimes: sharp peaks in the negative sign (λ_2_)­ρ domain, reflecting hydrogen bond stabilization; broad plateaus near zero, indicating the prevalence of weak, long-range dispersion; and positive shoulders, arising from steric hindrance. Notably, PTZ3, PTZ4 and PTZ9 exhibit more pronounced blue domains, in agreement with the shorter donor–acceptor hydrogen bond distances, while PTZ6 and PTZ8 present a predominance of green surfaces, implying that dispersion forces outweigh strong directional hydrogen bonding in these systems. The QTAIM analysis provides a rigorous topological framework for characterizing and quantifying interatomic interactions based on the electron density distribution. By analyzing bond critical point (BCP) parameters such as electron density [ρ­(r)], Laplacian [∇^2^ρ­(r)], kinetic [*G*(r)] and potential [*V*(r)] energy densities and Hessian eigenvalues (λ_1_, λ_2_), QTAIM enables an unambiguous distinction between covalent, ionic, hydrogen-bonding and van der Waals interactions (see the Figure 4). The QTAIM analysis of the O–H hydrogen bonds in PTZ1–PTZ10 shows bond distances in the range of 2.001–2.057 Å, with corresponding electron densities at the bond critical points [ρ­(r)] between 0.02214 and 0.02477 au, indicative of moderate hydrogen-bond strength. The Laplacian values [∇^2^ρ­(r) = *L*(r)] are consistently small and negative, ranging from −0.02150 au to −0.02396 au suggesting localized charge concentration in the bonding region and a slight partial covalent character.

**Figure 4:**
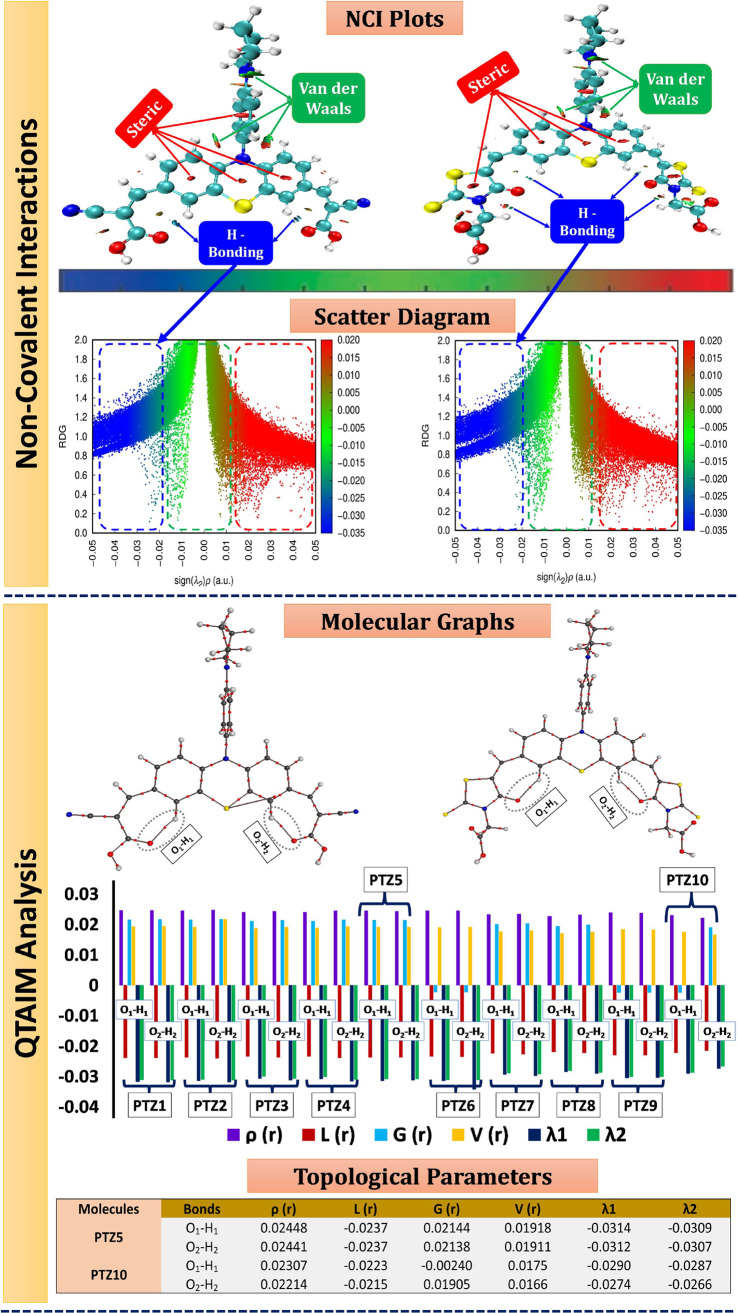
Noncovalent interaction (NCI) isosurfaces and corresponding reduced density gradient [RDG vs sign­(λ_2_)­ρ] scatter plots. Blue indicates strong attractive interactions, Green denotes weak van der Waals interactions and red corresponds to steric repulsion and QTAIM molecular graphs, showing bond paths, bond critical points, ring critical points and cage critical points obtained from electron density topology analysis.

The kinetic energy densities [*G*(*r*)] are positive from 0.01660 au to 0.02174 au and the potential energy densities [*V*(*r*)] are negative from −0.02384 au to −0.02150 au for all the PTZ molecules, confirming stabilizing interactions. The eigenvalues λ_1_ and λ_2_ are both negative, with magnitudes similar to each other for each bond, consistent with attractive interactions characteristic of hydrogen bonding. Among the series, PTZ2 exhibits the highest ρ­(r) value (0.02477 au) and one of the shortest O–H distances (2.005 Å), indicating a comparatively stronger interaction, whereas PTZ10 shows the longest O–H distance (2.057 Å) and one of the lowest ρ­(r) values (0.02214 au), reflecting a weaker interaction. QTAIM parameters indicate that the O–H interactions in the PTZ derivatives exhibit moderate electron density at the bond critical points. However, the negative Laplacian values suggest that these interactions are not purely closed-shell hydrogen bonds but instead possess partial covalent (shared-shell) character. Therefore, they are more appropriately described as hydrogen-bond-like interactions with mixed bonding characteristics.

### 3.8 Optoelectronic Properties: IPs, EAs and Charge Transport Reorganization Energies

The electronic properties of the PTZ derivatives were investigated by calculating ionization potentials (IPs), electron affinities (EAs) and reorganization energies for both hole (λ_hole_) and electron (λ_electron_) transport. The results are summarized in Table 4. The vertical IP values fall in the range of 6.68–7.31 eV, while the adiabatic IPs are slightly lower, between 6.32 and 6.96 eV. These relatively high values confirm the electron-donating nature of the phenothiazine core. Among the series, PTZ10, with the lowest adiabatic IP of 6.32 eV, emerges as the most favorable candidate for hole injection, as it requires less energy to remove an electron. Conversely, PTZ4 with the highest IP of 6.96 eV, shows comparatively weaker hole injection capability. These differences arise from the substituent-dependent modulation of electronic distribution across the PTZ framework. The electron affinities display complementary behavior, with vertical values ranging from −1.49 to −1.72 eV and adiabatic values from −1.74 to −1.94 eV. The stabilization observed in the adiabatic values reflects geometric relaxation upon electron addition. Notably, PTZ4 exhibits the largest EA (−1.94 eV), highlighting its strong electron-accepting capacity and suitability for electron injection processes. In contrast, PTZ10, with the lowest EA (−1.74 eV), appears less favorable for electron acceptance compared to other derivatives. The calculated reorganization energies provide further insight into charge-transport performance. For hole transport, λ_hole_ values range from 0.45 to 0.57 eV. PTZ7, with the lowest λ_hole_ of 0.45 eV, is predicted to exhibit efficient hole mobility, while PTZ3, with the highest λ_hole_ of 0.57 eV, is likely to suffer from reduced hole transport. For electron transport, λ_electron_ values span from 0.43 to 0.52 eV. PTZ2 and PTZ5, both with the lowest λ_electron_ of 0.43 eV, stand out as promising electron-transport materials. By comparison, PTZ8, PTZ9 and PTZ10, with λ_electron_ values in the range of 0.51–0.52 eV, show somewhat less favorable electron mobility. Importantly, in several molecules such as PTZ6 to PTZ10, the difference between λ_hole_ and λ_electron_ is relatively small, suggesting balanced ambipolar transport. Such balance is advantageous in optoelectronic applications where efficient recombination of charge carriers is required. Overall, these findings reveal distinct structure–property relationships across the PTZ series. PTZ10 and PTZ7 emerge as promising candidates for hole injection and transport, while PTZ4 stands out for its strong electron-injection ability. PTZ2 and PTZ5 exhibit the most favorable electron-transport characteristics due to their minimized reorganization energies. Molecules such as PTZ6 to PTZ10, which demonstrate balanced charge-transport properties, are well suited for ambipolar applications. The comparative analysis of light harvesting efficiency (LHE) and excited-state lifetime (τ) reveals a clear distinction in the performance of the PTZ derivatives.

**Table 4:** Calculated Ionization Potentials (IPs Vertical/Adiabatic in eV), Electron Affinities (EAs (Vertical/Adiabatic) in eV), Extraction Potentials (eV), Reorganization Energies (λ_hole_ and λ_electron_ in eV), Light Harvesting Efficiency (LHE), Radiative Lifetime (τ in ns) of the PTZ Molecules for Optoelctronic Applications

molecules	IP_(v)_	IP_(a)_	EA_(v)_	EA_(a)_	λ_hole_	λ_electron_	LHE	Δ*G*_inj_	τ
PTZ1	7.26	6.95	–1.66	–1.88	0.48	0.45	0.69	0.06	0.79
PTZ2	7.14	6.85	–1.67	–1.88	0.46	0.43	0.70	0.03	0.78
PTZ3	7.15	6.79	–1.62	–1.86	0.57	0.49	0.71	–0.01	0.78
PTZ4	7.31	6.96	–1.72	–1.94	0.54	0.47	0.69	0.10	0.82
PTZ5	7.04	6.74	–1.55	–1.77	0.50	0.43	0.72	–0.09	0.75
PTZ6	6.88	6.52	–1.58	–1.83	0.53	0.51	0.86	–0.22	0.48
PTZ7	6.72	6.44	–1.60	–1.83	0.45	0.49	0.86	–0.31	0.48
PTZ8	6.70	6.39	–1.57	–1.81	0.52	0.52	0.86	–0.26	0.46
PTZ9	6.88	6.55	–1.64	–1.88	0.52	0.52	0.86	–0.18	0.48
PTZ10	6.68	6.32	–1.49	–1.74	0.54	0.51	0.88	–0.33	0.47

Molecules PTZ1–PTZ5 display only moderate light absorption, with LHE values ranging between 0.69 and 0.72 and relatively short excited-state lifetimes of 0.43–0.49 ns. These characteristics suggest that, although these molecules are capable of absorbing photons, their excited states decay rapidly, leading to faster charge recombination and reduced potential for efficient charge transfer. In contrast, PTZ6–PTZ10 exhibit markedly superior properties, with significantly enhanced LHE values of 0.86–0.88. This combination reflects not only a stronger ability to capture and utilize incident photons but also improved stability of the excited states, both of which are critical for effective photoinduced electron transfer processes. Within this group, PTZ10 emerges as the promising derivative. It achieves the highest LHE (0.88), indicating exceptional light absorption capacity, while maintaining a stable τ value (0.47), ensuring sufficient excited-state persistence for efficient charge separation. The values range from 0.10 eV to −0.34 eV, where a negative value indicates a spontaneous and energetically favorable injection process. Specifically, molecules PTZ5 through PTZ10 exhibit negative Δ*G*_inj_ values, with PTZ10 showing the most negative value (−0.34 eV), suggesting it possesses the strongest driving force for efficient charge injection. In contrast, the positive values observed for PTZ1, PTZ2 and PTZ4 indicate a lack of sufficient energetic push, potentially hindering their performance in DSSCs. These results confirm that structural modifications in the PTZ series, particularly in PTZ10, successfully optimize the excited-state energy levels to facilitate rapid and significant electron injection into the semiconductor surface. Together, these features suggest that PTZ10 is a suitable candidate among the studied series for optoelectronic and photovoltaic applications. Collectively, the results highlight the tunability of PTZ derivatives for specific roles in optoelectronic properties.

### 3.9 MEP Analysis: Charge Distribution and Donor–Acceptor Features

The molecular electrostatic potential (MEP) surfaces of PTZ1-PTZ10 presented in the Supporting Information provide a comprehensive comparison of how structural modifications across the series influence charge distribution and potential reactive sites (see Figure 5). The red and blue regions, corresponding to the electron-rich and electron–deficient zones, reveal that while all PTZ derivatives display characteristics of donor–acceptor features, distinct variations arise depending on the nature of substituents. Across all molecules, the presence of both red and blue domains confirms their intrinsic donor–acceptor nature. Yet, the extent and sharpness of these regions vary significantly depending on the substituents introduced. Molecules PTZ1–PTZ5 exhibit relatively balanced electron density distributions, with the electrostatic potential values spanning from approximately −1.25 to −1.52 au in the electron-rich regions and +1.52 au in the electron-deficient regions.

**Figure 5:**
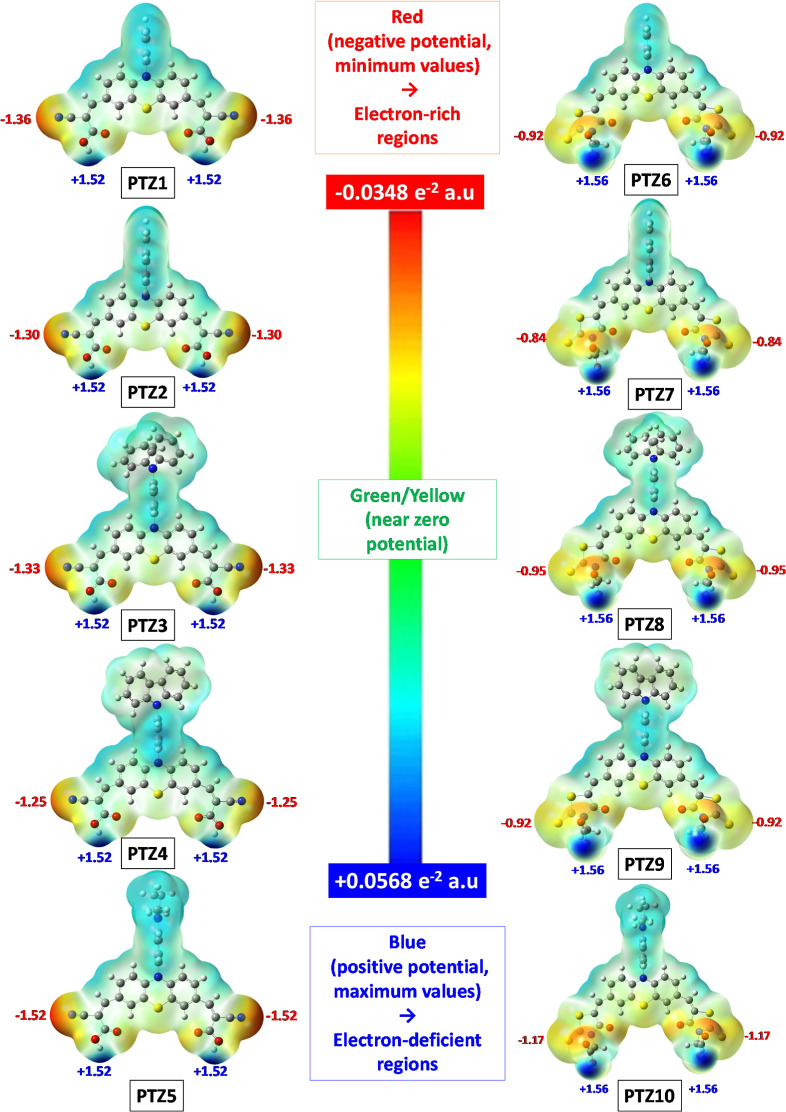
Electrostatic Potential surfaces of isolated PTZ5 and PTZ10. The red and blue represent regions of more negative and positive charges. The isodensity contours are 0.056 au.

In contrast, PTZ6–PTZ10 display reduced negative potentials (−0.84 to −1.17 au) and slightly more localized positive potential zones (+1.56 au), which indicate enhanced polarity and sharper charge separation compared to the earlier derivatives.

Among them, PTZ10 stands out with clear electron-rich and electron-deficient regions that are spatially well-separated, reflecting stronger intramolecular charge transfer and an improved donor–acceptor balance. This observation directly correlates with its high light-harvesting efficiency (0.88) and stable excited-state lifetime (0.51 ns), reinforcing its superior photoactive potential. Moreover, the increased polarity and distinct charge distribution in PTZ10 highlight its capacity to facilitate effective electron injection and charge transport, making it the most promising candidate of the series for optoelectronic and photovoltaic applications.

## 4 Conclusions

A systematic DFT and TD-DFT investigation of phenothiazine-based donor–π–acceptor derivatives (PTZ1–PTZ10) reveals clear structure–property relationships governing their optoelectronic behavior. Structural modifications through π-aryl substitution effectively tune molecular planarity, conjugation, and intramolecular charge transfer (ICT). Frontier molecular orbital analysis demonstrates efficient donor–acceptor separation across the series, with PTZ7, PTZ8 and PTZ10 exhibiting narrower band gaps, enhanced delocalization and improved electronic softness, all favorable for light harvesting and charge transport. Reorganization energy analysis identifies PTZ7 and PTZ10 as promising hole-transport materials, while PTZ2 and PTZ5 show superior electron-transport characteristics, indicating potential for balanced ambipolar performance. TD-DFT results confirm that PTZ6–PTZ10 display enhanced visible-light absorption, higher oscillator strengths and pronounced bathochromic shifts, supported by strong solvatochromic behavior. Excited-state analysis reveals small singlet–triplet energy gaps, suggesting efficient exciton utilization. Complementary QTAIM and NCI analyses highlight the role of hydrogen bonding, π–π interactions and dispersion forces in stabilizing molecular conformations and facilitating electronic communication. MEP results further confirm improved charge separation and polarity, particularly for PTZ10, which also exhibits the highest light-harvesting efficiency and favorable electron injection driving force. Overall, PTZ10 emerges as the most promising candidate, combining strong ICT, efficient light absorption, balanced charge transport and favorable energetic alignment for dye-sensitized solar cell applications. Despite these advantages, further structural optimization is required to extend absorption more deeply into the visible region. This work provides a robust theoretical framework for the rational design of phenothiazine-based chromophores for advanced optoelectronic and photovoltaic applications.

## Supplementary Material



## Notes

The Supporting Information presents NCI isosurfaces and RDG [sign­(λ_2_)­ρ] plots, together with QTAIM molecular graphs illustrating bond paths and critical points; DFT and TD-DFT calculations were performed to examine the electronic and excited-state properties of PTZ1–PTZ10; Frontier orbital energies and energy gaps were obtained using CAM-B3LYP, M06–2X, and B3LYP, and solvent-dependent absorption and emission data are included; Singlet (S_1_) and triplet (T_1_) excitation energies are also reported; QTAIM analysis provides key topological parameters at bond critical points, while noncovalent interactions are visualized through the corresponding plots and molecular graphs (PDF)
