# Supplementary material for: Unveiling the Electronic and Optoelectronic Behavior of Phenothiazine Derivatives through Theoretical Insights
Source: ACS Omega. 2026 Jun 24;11(26):38763–77. doi: 10.1021/acsomega.6c01934 (PMC13347371; doi:10.1021/acsomega.6c01934)
Supplement: Supplementary file 1 [file ao6c01934_si_001.pdf]

Supporting Information

Unveiling The Electronic and Optoelectronic Behaviour of Phenothiazine Derivatives Through Theoretical Insights

Murugesan Panneerselvam,[1,2] Anantha Narayanan Sri Gayathri,[3] Deepan Kumar Madhu,[4] Iravatham Rama,[3] Luciano T. Costa,[1*] Madhavan Jaccob[5*]

[1]MolMod-CS - Instituto de Química, Universidade Federal Fluminense, Campos Valonginho s/n, Centro, Niterói 24020-14, Rio de Janeiro, Brazil.

[2]Chemical Engineering Program (PEQ/COPPE), Federal University of Rio de Janeiro (UFRJ), Rio de Janeiro, 21941-594, RJ, Brazil

[3]PG and Research Department of Chemistry, Seethalakshmi Ramaswami College, Affiliated to Bharathidasan University, Tiruchirappalli 620 002, Tamil Nadu, India.

[4]Department of Chemistry, K. Ramakrishnan College of Technology, Samayapuram, Tiruchirapalli, Tamil Nadu 621112, India.

[5]Department of Chemistry & Computational Chemistry Laboratory, Loyola Institute of Frontier Energy (LIFE), Loyola College, Chennai 600 034, Tamil Nadu, India.

Email (s): ltcosta@id.uff.br & jaccob@loyolacollege.edu

Section 1. Methodology for Quantum Chemical descriptors

Global reactivity descriptors, namely electronegativity (χ), chemical potential (μ), global hardness (η), global softness (S) and electrophilicity index (ω), were calculated. These descriptors were derived based on Koopmans' theorem, wherein the frontier molecular orbital (FMO) energies play a critical role in governing molecular reactivity, electronic distribution, and kinetic stability.[1-5] Using ionization potential (IP)and electron affinity (EA):

electronegativity	χ=(IP+EA)/2	(S1)
Chemical Potential (μ)	$\mu = -\chi = (\partial E / \partial N)v(r)$	(S2)
Global Hardness (η)	η = (IP - EA) / 2	(S3)
Global Softness (S)	$S = 1 / \eta$	(S4)
Electrophilicity Index (ω)	$\omega = \mu^2 / (2\eta)$	(S5)

Here, IP and EA are estimated as the negative of HOMO and LUMO energies, respectively.

Table S1. Frontier Molecular Orbitals (FMOs) and their energy gap (E_g) values of PTZ derivatives obtained by Cam-B3LYP, M062X and B3LYP.

Molecules	HOMO-2	HOMO-1	HOMO	LUMO	LUMO+1	LUMO+2	E_g
**Cam-B3LYP**							
**PTZ1**	-8.65	-8.26	-7.06	-1.96	-1.49	-0.3	5.10
**PTZ2**	-8.23	-7.46	-7.02	-1.94	-1.47	-1.35	5.08
**PTZ3**	-8.2	-7.16	-7.01	-1.9	-1.45	-0.29	5.11
**PTZ4**	-7.74	-7.39	-7.16	-2	-1.56	-0.47	5.16
**PTZ5**	-8.1	-7.35	-6.88	-1.83	-1.36	-0.05	5.05
**PTZ6**	-7.76	-7.63	-6.74	-1.83	-1.54	-0.17	4.91
**PTZ7**	-7.62	-7.33	-6.64	-1.82	-1.5	-1.21	4.83
**PTZ8**	-7.6	-7.06	-6.61	-1.8	-1.48	-0.19	4.81
**PTZ9**	-7.67	-7.3	-6.77	-1.87	-1.58	-0.31	4.91
**PTZ10**	-7.54	-7.2	-6.57	-1.73	-1.43	-0.06	4.85
**M062X**							
**PTZ1**	-8.65	-8.26	-7.06	-1.96	-1.49	-0.3	5.10
**PTZ2**	-8.23	-7.46	-7.02	-1.94	-1.47	-1.35	5.08
**PTZ3**	-8.2	-7.16	-7.01	-1.9	-1.45	-0.29	5.11
**PTZ4**	-7.74	-7.39	-7.16	-2	-1.56	-0.47	5.16
**PTZ5**	-8.1	-7.35	-6.88	-1.83	-1.36	-0.05	5.05
**PTZ6**	-7.76	-7.63	-6.74	-1.83	-1.54	-0.17	4.91
**PTZ7**	-7.62	-7.33	-6.64	-1.82	-1.5	-1.21	4.83
**PTZ8**	-7.6	-7.06	-6.61	-1.8	-1.48	-0.19	4.81
**PTZ9**	-7.67	-7.3	-6.77	-1.87	-1.58	-0.31	4.91
**PTZ10**	-7.54	-7.2	-6.57	-1.73	-1.43	-0.06	4.85
**B3LYP**							
**PTZ1**	-8.58	-8.17	-6.96	-2.24	-1.76	-0.66	4.71
**PTZ2**	-8.15	-7.39	-6.91	-2.23	-1.74	-1.66	4.68
**PTZ3**	-8.12	-7.04	-6.92	-2.17	-1.73	-0.65	4.75
**PTZ4**	-7.67	-7.30	-7.08	-2.24	-1.82	-0.79	4.84
**PTZ5**	-8.01	-7.25	-6.78	-2.12	-1.63	-0.40	4.66
**PTZ6**	-7.65	-7.53	-6.63	-2.12	-1.82	-0.47	4.52
**PTZ7**	-7.51	-7.27	-6.51	-2.11	-1.77	-1.54	4.40
**PTZ8**	-7.49	-6.94	-6.49	-2.07	-1.75	-0.54	4.42
**PTZ9**	-7.58	-7.22	-6.68	-2.15	-1.86	-0.66	4.53
**PTZ10**	-7.44	-7.08	-6.43	-1.97	-1.69	-0.36	4.47
**PTZ1**	-8.58	-8.17	-6.96	-2.24	-1.76	-0.66	4.71

The frontier molecular orbital (FMO) analysis of PTZ1–PTZ10 derivatives provides a comprehensive understanding of how electronic structure is modulated by substitution and the choice of functional (Cam-B3LYP, M06-2X, and B3LYP). Overall, the data reveal consistent orbital trends across all methods, with subtle but meaningful differences in absolute energies and energy gaps (E_g), which are critical for optoelectronic and DSSC-related applications. Across all functionals, the HOMO energies gradually increase (become less negative) from PTZ1 to PTZ10. For example, at the Cam-B3LYP level, the HOMO rises from −7.06 eV (PTZ1) to −6.57 eV (PTZ10), indicating enhanced electron-donating ability with structural

modification. A similar trend is observed for M06-2X (identical values here) and B3LYP (−6.96 to −6.43 eV). This upward shift in HOMO suggests that later PTZ derivatives (particularly PTZ7–PTZ10) are more prone to oxidation, which is advantageous for efficient dye regeneration in photovoltaic systems. The HOMO−1 and HOMO−2 levels follow the same trend, indicating a systematic destabilization of occupied orbitals due to substituent effects and increased conjugation. In contrast, the LUMO energies also show a slight upward trend (less negative values), but the variation is smaller compared to the HOMO. At the Cam-B3LYP level, LUMO energies range from −2.00 eV (PTZ4) to −1.73 eV (PTZ10), while B3LYP predicts more stabilized LUMO levels (−2.24 to −1.97 eV). This consistent stabilization by B3LYP reflects its known tendency to underestimate band gaps due to self-interaction error and lack of long-range correction. Importantly, the relatively small variation in LUMO energies suggests that acceptor strength is less sensitive to substitution than donor strength in these PTZ systems. The HOMO–LUMO energy gap (Eg) shows a clear decreasing trend from PTZ1 to PTZ10 for all methods. Using Cam-B3LYP, Eg decreases from 5.10 eV (PTZ1) to 4.81 eV (PTZ8), with a slight fluctuation thereafter. M06-2X reproduces identical values in this dataset, indicating either computational consistency or reporting duplication. B3LYP yields systematically lower E_g values (4.71–4.40 eV), again due to its intrinsic functional limitations. The narrowing of E_g in PTZ6–PTZ10 indicates improved electronic delocalization and stronger intramolecular charge transfer (ICT), which is beneficial for light absorption and red-shifted optical transitions. Among the derivatives, PTZ7 and PTZ8 exhibit the smallest energy gaps (4.83 and 4.81 eV at Cam-B3LYP; 4.40 and 4.42 eV at B3LYP), suggesting that these molecules possess the most favorable electronic structures for optoelectronic applications. Their relatively high HOMO levels combined with moderately low LUMO levels facilitate efficient charge separation and transfer. Conversely, PTZ4 shows the largest E_g (5.16 eV), attributed to its deeper HOMO (−7.16 eV) and relatively stabilized LUMO (−2.00 eV), indicating weaker conjugation or less effective donor–acceptor interaction. Higher virtual orbitals (LUMO+1 and LUMO+2) also provide insight into excited-state accessibility. The relatively small energy spacing between LUMO and LUMO+1 (typically ~0.4–0.5 eV) suggests the possibility of multiple low-lying excited states contributing to absorption spectra. However, the larger variation in LUMO+2 (e.g., −1.21 eV for PTZ7 vs −0.05 eV for PTZ5 at Cam-B3LYP) indicates structural sensitivity in higher excited states, which may influence spectral broadening and charge-transfer pathways. Importantly, the near-identical values reported for Cam-B3LYP and M06-2X across all orbitals and molecules are unusual, as these functionals typically produce distinct energetic profiles. This strongly suggests either a

duplication error or that the same dataset has been inadvertently reported under both methods. This point should be carefully verified, as it affects the reliability of methodological comparisons. In summary, the FMO analysis demonstrates that (i) substitution in PTZ derivatives primarily modulates the HOMO level, enhancing donor strength; (ii) LUMO levels are comparatively less sensitive but still contribute to tuning E_g; (iii) the energy gap decreases systematically, favoring improved ICT and optical properties in later derivatives; and (iv) B3LYP underestimates E_g relative to long-range corrected functionals like Cam-B3LYP. Among the studied systems, PTZ7–PTZ10 emerge as the most promising candidates for applications requiring efficient charge transfer and reduced band gaps, although methodological consistency between functionals must be confirmed for robust conclusions.

Table S2. Calculated electronic properties [absorption wavelengths in nm and oscillator strengths(f_0)] of PTZ Molecules in Methanol(ε=33) medium.

Molecules	λ_1	f_0	λ_2	f_0	λ_3	f_0	λ_4	f_0
PTZ1	440.4	0.911	351.8	0.559	315.9	0.649	293.7	0.608
PTZ2	441.7	0.911	355.0	0.814	315.8	0.467	294.4	0.623
PTZ3	436.7	0.951	352.8	0.682	315.1	0.626	293.2	0.552
PTZ4	432.2	0.929	350.4	0.608	314.4	0.638	293.0	0.319
PTZ5	445.9	0.961	355.0	0.627	317.0	0.618	294.1	0.595
PTZ6	451.2	1.188	389.1	1.427	334.6	0.386	320.9	0.252
PTZ7	462.5	1.185	393.3	1.462	338.4	0.508	323.9	0.264
PTZ8	464.2	1.218	393.6	1.373	337.5	0.494	324.8	0.198
PTZ9	452.5	1.205	389.6	1.393	335.7	0.446	322.1	0.261
PTZ10	458.4	1.227	392.8	1.412	335.2	0.398	321.2	0.237

Table S3. Calculated electronic properties [absorption wavelengths in nm and oscillator strengths(f_0)] of PTZ Molecules in Acetonitrile(ε=36) medium.

Molecules	λ_1	f_0	λ_2	f_0	λ_3	f_0	λ_4	f_0
PTZ1	440.6	0.913	352.0	0.561	315.9	0.650	293.7	0.607
PTZ2	441.9	0.913	355.2	0.815	317.8	0.198	294.4	0.622
PTZ3	436.0	0.953	353.0	0.682	315.1	0.626	293.2	0.547
PTZ4	432.3	0.931	350.5	0.610	314.1	0.638	292.0	0.591
PTZ5	446.1	0.963	355.2	0.628	317.0	0.618	294.1	0.594
PTZ6	451.4	1.189	389.3	1.429	334.6	0.384	320.9	0.251
PTZ7	462.7	1.186	393.5	1.463	338.4	0.507	323.9	0.262
PTZ8	464.4	1.219	393.8	1.374	337.6	0.493	324.9	0.194
PTZ9	452.7	1.206	389.9	1.394	335.7	0.444	332.1	0.260
PTZ10	458.6	1.228	393.0	1.413	335.2	0.397	321.3	0.237

Table S4. Calculated electronic properties [absorption wavelengths in nm and oscillator strengths(f_0)] of PTZ Molecules in Dichloromethane (ε=9.1) medium.

Molecules	λ_1	f_0	λ_2	f_0	λ_3	f_0	λ_4	f_0
PTZ1	437.1	0.867	348.9	0.523	315.0	0.640	293.6	0.637
PTZ2	438.5	0.868	352.4	0.788	316.3	0.363	294.3	0.653
PTZ3	433.5	0.905	350.1	0.658	314.3	0.617	292.1	0.708
PTZ4	428.8	0.881	347.5	0.582	313.5	0.628	291.9	0.574
PTZ5	442.6	0.917	352.2	0.594	316.2	0.611	293.9	0.593
PTZ6	447.6	1.163	384.0	1.385	333.9	0.413	320.1	0.268
PTZ7	458.9	1.157	388.6	1.440	337.5	0.537	322.9	0.306
PTZ8	460.6	1.189	388.7	1.348	336.8	0.525	323.3	0.284
PTZ9	448.7	1.177	384.6	1.368	334.9	0.475	321.2	0.280
PTZ10	454.9	1.202	387.9	1.377	334.5	0.427	320.1	0.234

Table S5. Calculated electronic properties [absorption wavelengths in nm and oscillator strengths(f_0)] of PTZ Molecules in Chloroform (ε=4.8) medium.

Molecules	λ_1	f_0	λ_2	f_0	λ_3	f_0	λ_4	f_0
PTZ1	433.5	0.817	345.9	0.481	314.0	0.622	293.4	0.675
PTZ2	435.0	0.821	349.6	0.752	315.0	0.48	294.1	0.691
PTZ3	430.0	0.854	347.2	0.627	313.4	0.601	292.2	0.654
PTZ4	425.3	0.829	344.5	0.550	312.5	0.611	291.8	0.650
PTZ5	439.0	0.868	349.2	0.555	315.4	0.597	294.0	0.595
PTZ6	443.6	1.132	378.5	1.331	333.1	0.443	319.2	0.290
PTZ7	455.0	1.124	383.7	1.407	336.6	0.568	321.7	0.351
PTZ8	456.6	1.154	383.5	1.313	335.9	0.557	322.0	0.343
PTZ9	444.7	1.143	379.2	1.334	334.1	0.507	320.2	0.307
PTZ10	451.1	1.171	382.6	1.330	333.8	0.456	324.8	0.112

Table S6. Calculated electronic properties [absorption wavelengths in nm and oscillator strengths(f_0)] of PTZ Molecules in Toluene(ε=2.4) medium.

**Molecules**	λ_1	f_0	λ_2	f_0	λ_3	f_0	λ_4	f_0
PTZ1	426.6	0.716	340.3	0.394	312.1	0.560	292.9	0.768
PTZ2	428.2	0.724	344.4	0.663	312.9	0.495	293.5	0.785
PTZ3	423.3	0.750	342.0	0.555	311.4	0.542	291.7	0.733
PTZ4	418.5	0.723	339.1	0.477	310.5	0.551	291.5	0.730
PTZ5	432.0	0.766	343.7	0.469	313.6	0.546	293.3	0.731
PTZ6	435.8	1.062	368.0	1.193	331.6	0.500	317.3	0.350
PTZ7	447.2	1.050	374.2	1.295	334.7	0.625	319.3	0.460
PTZ8	448.7	1.075	373.6	1.194	334.2	0.618	319.4	0.446
PTZ9	436.7	1.067	369.0	1.233	332.4	0.567	318.0	0.379
PTZ10	443.3	1.100	372.7	1.027	332.3	0.511	319.8	0.328

Table S7. Calculated electronic properties [emission wavelengths in nm and oscillator strengths(f_0)] of PTZ Molecules in Methanol(ε=33) medium.

**Molecules**	λ_1	f_0	λ_2	f_0	λ_3	f_0	λ_4	f_0
PTZ1	562.4	0.648	398.3	0.411	337.3	0.855	323.1	0.980
PTZ2	559.0	0.636	399.0	0.536	337.6	0.495	332.2	0.763
PTZ3	564.5	0.655	399.7	0.472	337.8	0.849	323.2	0.971
PTZ4	559.9	0.634	397.8	0.442	337.0	0.852	323.2	0.988
PTZ5	568.2	0.674	400.6	0.452	338.4	0.841	323.2	0.966
PTZ6	575.6	0.898	434.8	1.008	369.2	0.914	364.4	0.0003
PTZ7	572.7	0.885	435.1	1.137	369.6	0.922	350.3	0.692
PTZ8	577.4	0.924	436.2	1.057	368.6	0.911	364.1	0.001
PTZ9	573.1	0.892	434.3	1.050	369.2	0.916	364.7	0.0001
PTZ10	583.5	0.919	438.5	1.037	369.4	0.894	363.7	0.0002

Table S8. Calculated electronic properties [emission wavelengths in nm and oscillator strengths(f_0)] of PTZ Molecules in Acetonitrile(ε=36) medium.

Molecules	λ_1	f_0	λ_2	f_0	λ_3	f_0	λ_4	f_0
PTZ1	562.6	0.650	398.5	0.412	337.3	0.860	323.1	0.975
PTZ2	559.2	0.637	399.1	0.537	337.7	0.491	332.2	0.752
PTZ3	564.7	0.657	399.9	0.473	337.9	0.853	323.2	0.966
PTZ4	560.1	0.636	398.0	0.443	337.1	0.857	323.2	0.983
PTZ5	568.4	0.676	400.8	0.453	338.4	0.845	323.2	0.961
PTZ6	575.9	0.899	435.1	1.010	369.2	0.912	364.3	0.0003
PTZ7	572.9	0.886	435.4	1.139	369.6	0.921	350.3	0.690
PTZ8	577.7	0.926	436.5	1.058	368.6	0.909	364.1	0.001
PTZ9	573.3	0.893	434.6	1.051	369.2	0.915	364.6	0.0001
PTZ10	583.7	0.92	438.8	1.039	369.5	0.893	363.7	0.0002

Table S9. Calculated electronic properties [emission wavelengths in nm and oscillator strengths(f_0)] of PTZ Molecules in Dichloromethane (ε=9.1) medium.

Molecules	λ_1	f_0	λ_2	f_0	λ_3	f_0	λ_4	f_0
PTZ1	557.3	0.611	394.8	0.385	336.0	0.741	322.7	1.108
PTZ2	554.3	0.601	395.8	0.515	337.4	0.187	332.8	1.038
PTZ3	559.6	0.618	396.4	0.451	336.5	0.738	322.8	1.088
PTZ4	554.9	0.596	394.3	0.422	335.7	0.727	322.9	1.119
PTZ5	563.3	0.638	397.3	0.428	337.2	0.741	322.9	1.078
PTZ6	570.0	0.867	429.0	0.955	368.0	0.927	366.7	0.020
PTZ7	567.5	0.855	429.9	1.096	368.3	0.956	367.0	0.0005
PTZ8	572.0	0.893	430.6	1.016	367.4	0.902	366.3	0.035
PTZ9	567.3	0.859	428.5	1.008	367.9	0.948	367.1	0.003
PTZ10	578.0	0.888	433.0	0.989	368.3	0.918	365.8	0.009

Table S10. Calculated electronic properties [emission wavelengths in nm and oscillator strengths(f_0)] of PTZ Molecules in Chloroform (ε=4.8) medium.

Molecules	λ_1	f_0	λ_2	f_0	λ_3	f_0	λ_4	f_0
PTZ1	551.9	0.571	391.0	0.355	334.7	0.592	322.1	1.269
PTZ2	549.4	0.563	392.4	0.489	332.4	1.053	330.3	0.103
PTZ3	554.5	0.578	392.8	0.428	335.3	0.594	322.2	1.236
PTZ4	549.6	0.555	390.6	0.398	334.4	0.564	322.2	1.285
PTZ5	558.0	0.598	393.8	0.400	336.0	0.612	322.2	1.218
PTZ6	563.9	0.831	423.0	0.890	369.2	0.009	366.6	0.973
PTZ7	562.0	0.820	424.4	1.042	369.3	0.001	366.9	0.991
PTZ8	566.1	0.857	424.8	0.965	368.8	0.009	366.0	0.969
PTZ9	561.2	0.822	422.4	0.955	369.6	0.001	366.5	0.985
PTZ10	572.1	0.853	427.3	0.928	368.3	0.048	366.9	0.906

Table S11. Calculated electronic properties [emission wavelengths in nm and oscillator strengths(f_0)] of PTZ Molecules in Toluene(ε=2.4) medium.

Molecules	λ_1	f_0	λ_2	f_0	λ_3	f_0	λ_4	f_0
PTZ1	541.7	0.276	384.2	0.295	332.9	0.289	319.7	1.583
PTZ2	539.9	0.488	386.2	0.43	330.4	0.671	323.5	0.106
PTZ3	544.6	0.499	386.4	0.376	333.2	0.266	319.9	1.526
PTZ4	539.6	0.475	383.9	0.347	332.7	0.250	319.9	1.590
PTZ5	547.8	0.518	387.3	0.343	334.1	0.335	320.0	1.503
PTZ6	552.3	0.755	411.9	0.742	374.1	0.001	363.5	1.042
PTZ7	551.3	0.746	414.2	0.910	373.9	0.0003	363.9	1.047
PTZ8	554.8	0.779	414.2	0.840	373.5	0.001	363.0	1.037
PTZ9	549.6	0.743	411.4	0.826	374.5	0.0004	363.4	1.045
PTZ10	560.6	0.778	416.7	0.789	372.8	0.001	364.0	1.015

Table S12. Solvent-dependent variation of the calculated absorption and emission maxima (λ_{max} in nm) of PTZ1–PTZ10 in solvents of varying dielectric constants obtained using TD-DFT calculations.

Molecules	Acetonitrile (ε=36)	Methanol (ε=33)	Dichloromethane (ε=9.1)	Chloroform (ε=4.8)	Toluene (ε=2.4)
**Absorption**					
**PTZ1**	440.6	440.4	437.1	433.5	426.6
**PTZ2**	441.9	441.7	438.5	435.0	428.2
**PTZ3**	436.0	436.7	433.5	430.0	423.3
**PTZ4**	432.3	432.2	428.8	425.3	418.5
**PTZ5**	446.1	445.9	442.6	439.0	432.0
**PTZ6**	451.4	451.2	447.6	443.6	435.8
**PTZ7**	462.7	462.5	458.9	455.0	447.2
**PTZ8**	464.4	464.2	460.6	456.6	448.7
**PTZ9**	452.7	452.5	448.7	444.7	436.7
**PTZ10**	458.6	458.4	454.9	451.1	443.3
**Emission**					
**PTZ1**	562.6	562.4	557.3	551.9	541.7
**PTZ2**	559.2	559.0	554.3	549.4	539.9
**PTZ3**	564.7	564.5	559.6	554.5	544.6
**PTZ4**	560.1	559.9	554.9	549.6	539.6
**PTZ5**	568.4	568.2	563.3	558.0	547.8
**PTZ6**	575.9	575.6	570.0	563.9	552.3
**PTZ7**	572.9	572.7	567.5	562.0	551.3
**PTZ8**	577.7	577.4	572.0	566.1	554.8
**PTZ9**	573.3	573.1	567.3	561.2	549.6
**PTZ10**	583.7	583.5	578.0	572.1	560.6

Table S13. Singlet (S_1) and triplet (T_1) excitation energies (in eV) were calculated using TD-DFT at different functionals (CAM-B3LYP, M06, M06-2X, PBE and PBE0).

**Molecules**	**Cam-B3LYP**			**M06**			**M06-2X**			**PBE**			**PBE0**		
	S_1	T_1	ΔE_{ST}	S_1	T_1	ΔE_{ST}	S_1	T_1	ΔE_{ST}	S_1	T_1	ΔE_{ST}	S_1	T_1	ΔE_{ST}
PTZ1	3.001	2.28	0.721	2.516	2.015	0.501	2.954	2.344	0.61	1.95	1.543	0.407	2.526	1.978	0.548
PTZ2	2.99	2.27	0.72	2.509	2.004	0.505	2.942	2.331	0.611	1.951	1.534	0.417	2.52	1.967	0.553
PTZ3	3.021	2.295	0.726	2.535	2.036	0.499	2.971	2.354	0.617	1.705	1.55	0.155	2.542	1.992	0.55
PTZ4	3.057	2.323	0.734	2.562	2.067	0.495	3.006	2.389	0.617	1.828	1.579	0.249	2.57	2.023	0.547
PTZ5	2.966	2.248	0.718	2.493	1.99	0.503	2.918	2.303	0.615	1.846	1.514	0.332	2.502	1.944	0.558
PTZ6	2.955	2.133	0.822	2.441	1.935	0.506	2.9	2.21	0.69	1.806	1.436	0.37	2.432	1.867	0.565
PTZ7	2.877	2.096	0.781	2.37	1.868	0.502	2.821	2.165	0.656	1.772	1.383	0.389	2.366	1.813	0.553
PTZ8	2.867	2.09	0.777	2.366	1.869	0.497	2.811	2.157	0.654	1.765	1.375	0.39	2.357	1.805	0.552
PTZ9	2.947	2.133	0.814	2.428	1.919	0.509	2.893	2.211	0.682	1.797	1.429	0.368	2.421	1.862	0.559
PTZ10	3.001	2.28	0.721	2.399	1.897	0.502	2.849	2.179	0.67	1.783	1.398	0.385	2.389	1.833	0.556

Table S14. Topological parameters obtained from QTAIM analysis at the bond critical points for PTZ derivatives (PTZ1 to PTZ10). *ρ*(r): electron density; *G*(r): kinetic energy density; *V*(r): potential energy density; *L*(r): total energy density (*L*(r) = *G*(r) + *V*(r)); λ_1 and λ_2: negative eigenvalues of the Hessian matrix of ρ(r), representing the curvature perpendicular to the bond path.

Molecules	Bonds	Distance	ρ (r)	L (r)	G (r)	V (r)	λ1	λ2
PTZ1	O-H	2.001	0.02463	-0.02384	0.02159	0.01934	-0.03167	-0.03108
	O-H	2.003	0.02463	-0.02384	0.02165	0.01941	-0.03177	-0.03124
PTZ2	O-H	2.001	0.02451	-0.02373	0.02147	0.01921	-0.03141	-0.03091
	O-H	2.005	0.02477	-0.02396	0.02174	0.02174	-0.03188	-0.03128
PTZ3	O-H	2.008	0.02402	-0.02333	0.02101	0.01869	-0.03059	-0.02986
	O-H	2.016	0.02441	-0.02365	0.02138	0.01911	-0.03125	-0.03065
PTZ4	O-H	2.005	0.02410	-0.02341	0.02110	0.01879	-0.03074	-0.02997
	O-H	2.015	0.02455	-0.02377	0.02151	0.01926	-0.03151	-0.03091
PTZ6	O-H	2.007	0.02443	-0.02344	-0.00221	0.01901	-0.03136	-0.03101
	O-H	2.006	0.02449	-0.02350	-0.00220	0.01908	-0.03417	-0.03114
PTZ7	O-H	2.027	0.02324	-0.02240	0.02004	0.01769	-0.02937	-0.02891
	O-H	2.03	0.02349	-0.02263	0.02031	0.01799	-0.02976	-0.02917
PTZ8	O-H	2.033	0.02272	-0.02193	0.01952	0.01711	-0.02845	-0.02815
	O-H	2.04	0.02309	-0.02225	0.01988	0.01751	-0.02907	-0.02871
PTZ9	O-H	2.019	0.02390	-0.02299	-0.00227	0.01843	-0.03048	-0.02997
	O-H	2.018	0.02380	-0.02288	-0.00229	0.01829	-0.03030	-0.03001

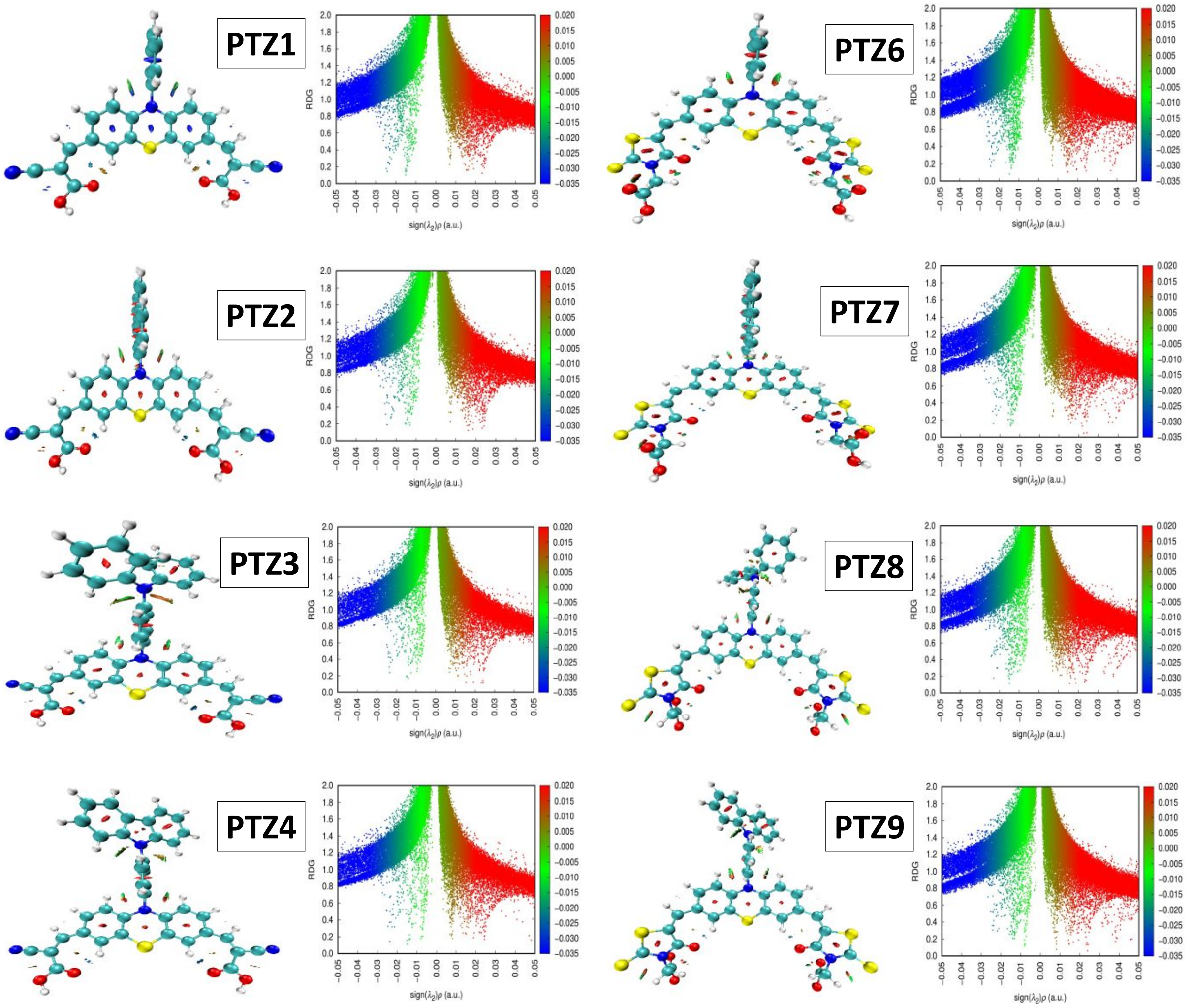

Figure S1. Noncovalent interaction (NCI) isosurfaces and corresponding reduced density gradient [RDG vs sign(λ_2)ρ] scatter plots. Blue indicates strong attractive interactions, Green denotes weak van der Waals interactions and red corresponds to steric repulsion.

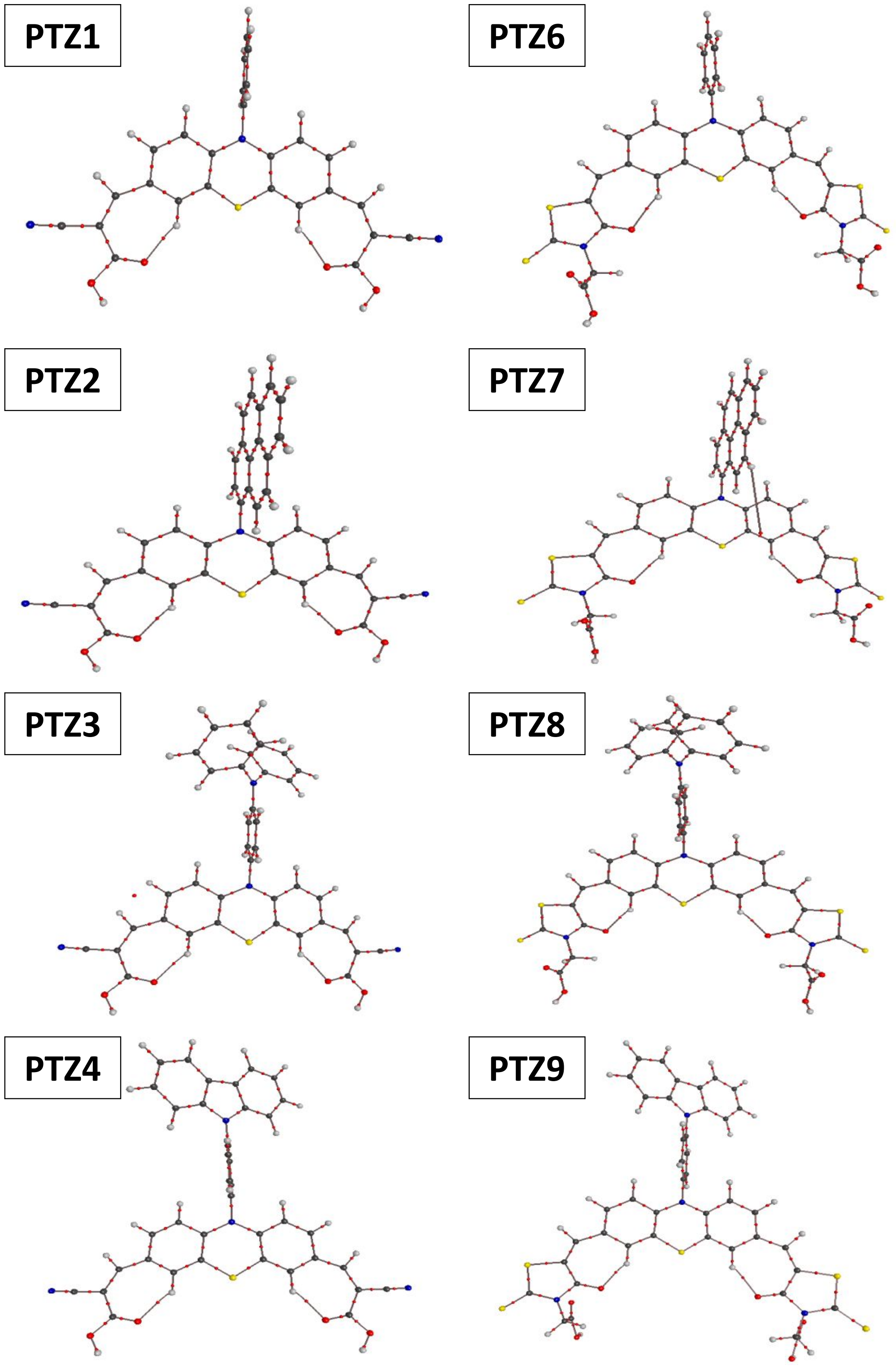

Figure S2. QTAIM molecular graphs showing bond paths, bond critical points, ring critical points and cage critical points obtained from electron density topology analysis.

References

1. Parr, R. G.; Donnelly, R. A.; Levy, M.; Palke, W. E. Electronegativity: The Density Functional Viewpoint. *J. Chem. Phys.* **1978**, *68*, 3801–3807.
2. Parr, R. G.; Pearson, R. G. Absolute Hardness: Companion Parameter to Absolute Electronegativity. *J. Am. Chem. Soc.* **1983**, *105*, 7512–7516.
3. Heinrich, N.; Koch, W.; Frenking, G. On the Use of Koopmans' Theorem to Estimate Negative Electron Affinities. *Chem. Phys. Lett.* **1986**, *124*, 20–25.
4. Madhu, D. K., & Madhavan, J. (2018). Quantum chemical analysis of electronic structure and bonding aspects of choline-based ionic liquids. Journal *of Molecular Liquids*, *249*, 637-649.
5. Kumar, M. D., & Jaccob, M. (2021). The keto–enol tautomerization of ethyl acetoacetate in choline ionic liquids: the role of cation and anion in switching the tautomeric equilibrium. *Physical Chemistry Chemical Physics*, *23*(12), 7386-7397.

Cartesian Coordinates of Ground State Optimized Structures

PTZ1

6	-3.665444000	-0.240572000	0.351017000
6	-2.485911000	-0.741116000	-0.212310000
6	-1.294805000	-0.057759000	-0.102600000
6	-1.206232000	1.133567000	0.630305000
6	-2.381406000	1.643709000	1.180731000
6	-3.575961000	0.977660000	1.030388000
6	1.250839000	1.148667000	0.694342000
6	1.395358000	-0.036135000	-0.039944000
6	2.602583000	-0.697620000	-0.096891000
1	2.672095000	-1.616981000	-0.654514000
6	3.743361000	-0.183536000	0.530260000
6	3.597945000	1.023221000	1.220248000
6	2.386777000	1.669382000	1.312989000
1	-2.511638000	-1.667195000	-0.762423000
1	-2.364449000	2.572944000	1.726712000
1	-4.467162000	1.409403000	1.467032000
1	4.457353000	1.462185000	1.710242000
1	2.326016000	2.591886000	1.867106000
7	0.014474000	1.798986000	0.797641000
16	0.077475000	-0.650605000	-1.027769000
6	-0.007615000	3.100826000	1.404887000
6	0.020571000	4.220678000	0.593527000
6	-0.052350000	3.237230000	2.784105000
6	0.004876000	5.483981000	1.163141000
1	0.055851000	4.095239000	-0.480377000
6	-0.068553000	4.499962000	3.349764000
1	-0.073786000	2.352577000	3.407294000
6	-0.039587000	5.624435000	2.539522000
1	0.027592000	6.359485000	0.528386000
1	-0.103172000	4.606651000	4.425703000
1	-0.051769000	6.610871000	2.983349000
6	-4.985104000	-0.834883000	0.311544000
6	5.073059000	-0.756320000	0.552011000
1	5.763263000	-0.110177000	1.083997000
1	-5.714477000	-0.187223000	0.786233000
6	-5.547545000	-1.983742000	-0.136741000
6	5.679566000	-1.883719000	0.106805000
6	-6.957045000	-2.115177000	0.095310000
6	-4.894821000	-3.123236000	-0.817938000
7	-8.083621000	-2.177420000	0.297055000
8	-5.749974000	-4.137635000	-1.001939000
8	-3.750297000	-3.184493000	-1.181456000
1	-5.264511000	-4.843782000	-1.452759000
6	5.088312000	-3.012561000	-0.644839000
6	7.075536000	-1.999925000	0.416353000
7	8.189455000	-2.052083000	0.681159000
8	3.970551000	-3.078422000	-1.083626000
8	5.969703000	-4.009239000	-0.801242000
1	5.526543000	-4.708166000	-1.304192000

PTZ2

6	1.899127000	-3.618848000	-0.364135000
6	2.408319000	-2.411718000	0.129015000
6	1.659317000	-1.255861000	0.102889000
6	0.380427000	-1.231254000	-0.470486000
6	-0.136851000	-2.434298000	-0.948375000
6	0.602935000	-3.592601000	-0.887159000
6	0.227418000	1.222677000	-0.514744000
6	1.479415000	1.434701000	0.076385000
6	2.060511000	2.683880000	0.089049000
1	3.028778000	2.808471000	0.544824000
6	1.403404000	3.795886000	-0.448834000
6	0.146640000	3.575619000	-1.020246000
6	-0.427384000	2.326272000	-1.059656000
1	3.394228000	-2.389122000	0.563141000
1	-1.128991000	-2.465869000	-1.368331000
1	0.164873000	-4.506034000	-1.267888000
1	-0.394867000	4.408073000	-1.450441000
1	-1.397706000	2.207106000	-1.513859000
7	-0.362202000	-0.047594000	-0.551145000
16	2.296656000	0.146625000	0.950202000
6	2.558479000	-4.906959000	-0.402254000
6	1.874455000	5.163963000	-0.490396000
1	1.177480000	5.796319000	-1.029927000
1	1.914335000	-5.656533000	-0.849308000
6	3.760324000	-5.423686000	-0.046509000
6	2.940925000	5.864707000	-0.033189000
6	3.944659000	-6.817172000	-0.332776000
6	4.901814000	-4.739813000	0.597823000
7	4.051106000	-7.931276000	-0.580387000
8	5.958847000	-5.554900000	0.713034000
8	4.930948000	-3.603683000	0.991300000
1	6.663069000	-5.050482000	1.145306000
6	4.087487000	5.380060000	0.765536000
6	2.944843000	7.263605000	-0.351579000
7	2.907074000	8.376099000	-0.624724000
8	4.284541000	4.251594000	1.132216000
8	4.931882000	6.377071000	1.061044000
1	5.654019000	5.996821000	1.582086000
6	-1.738199000	-0.134248000	-0.951344000
6	-2.062496000	-0.231273000	-2.294631000
6	-2.734686000	-0.105875000	0.025657000
6	-3.379818000	-0.307598000	-2.697734000
1	-1.265705000	-0.245773000	-3.027060000
6	-4.087771000	-0.180915000	-0.388056000
6	-2.445824000	0.002205000	1.427034000
6	-4.410018000	-0.284582000	-1.760824000
1	-3.619725000	-0.383484000	-3.750423000
6	-5.127634000	-0.148762000	0.581448000
6	-3.435008000	0.034471000	2.338563000
1	-1.413332000	0.057816000	1.741084000
6	-5.789163000	-0.359192000	-2.145242000
6	-6.478869000	-0.224003000	0.173150000
6	-4.813494000	-0.038886000	1.954744000
1	-3.199954000	0.116814000	3.392379000
6	-6.771174000	-0.331108000	-1.225934000
1	-6.024398000	-0.437722000	-3.199142000
6	-7.480884000	-0.188965000	1.138770000
6	-5.845767000	-0.005589000	2.887127000
1	-7.808710000	-0.387139000	-1.530559000
6	-7.165640000	-0.080485000	2.480772000
1	-8.516132000	-0.246455000	0.827098000
1	-5.604601000	0.079800000	3.939128000
1	-7.957015000	-0.053591000	3.217779000

PTZ3

6	-2.984754000	-3.558985000	-0.717571000
6	-3.281416000	-2.405602000	0.017805000
6	-2.568848000	-1.240971000	-0.164624000
6	-1.554323000	-1.152132000	-1.127890000
6	-1.249046000	-2.300829000	-1.856049000
6	-1.946150000	-3.468662000	-1.650054000
6	-1.440652000	1.294201000	-1.072871000
6	-2.431297000	1.441312000	-0.092948000
6	-3.011402000	2.665844000	0.158411000
1	-3.772323000	2.740752000	0.917456000
6	-2.605294000	3.815039000	-0.529888000
6	-1.606796000	3.657913000	-1.496679000
6	-1.038065000	2.434923000	-1.765738000

1	-4.062143000	-2.433764000	0.759678000
1	-0.458491000	-2.279852000	-2.588549000
1	-1.683292000	-4.338790000	-2.237716000
1	-1.270106000	4.520464000	-2.057143000
1	-0.271355000	2.363958000	-2.520222000
7	-0.867103000	0.046431000	-1.349370000
16	-2.866447000	0.090317000	0.945334000
6	0.342266000	0.016840000	-2.123244000
6	1.560868000	0.008422000	-1.467540000
6	0.322477000	0.014857000	-3.509359000
6	2.743738000	-0.001890000	-2.180445000
1	1.579935000	0.001589000	-0.385737000
6	1.501054000	0.013006000	-4.228932000
1	-0.625477000	0.031395000	-4.031758000
6	2.735401000	0.006424000	-3.575432000
1	3.686259000	-0.014934000	-1.652548000
1	1.468454000	0.025227000	-5.308904000
6	-3.637350000	-4.848088000	-0.636374000
6	-3.105012000	5.164224000	-0.368775000
1	-2.669262000	5.828057000	-1.107964000
1	-3.234433000	-5.532028000	-1.375880000
6	-4.612784000	-5.427469000	0.106096000
6	-3.957538000	5.819982000	0.456167000
6	-4.921635000	-6.786760000	-0.232978000
6	-5.386233000	-4.847412000	1.225364000
7	-5.138209000	-7.868860000	-0.542918000
8	-6.279412000	-5.723089000	1.706290000
8	-5.266787000	-3.743896000	1.688743000
1	-6.745130000	-5.285921000	2.434016000
6	-4.717292000	5.286203000	1.607013000
6	-4.123524000	7.219842000	0.189356000
7	-4.226834000	8.334612000	-0.056643000
8	-4.723590000	4.150030000	2.000917000
8	-5.432207000	6.247154000	2.207730000
1	-5.899780000	5.834879000	2.948694000
7	3.935199000	0.017054000	-4.297970000
6	4.037234000	-0.631602000	-5.553927000
6	3.515917000	-1.907541000	-5.738755000
6	4.682924000	-0.004535000	-6.613908000
6	3.628709000	-2.536569000	-6.965351000
1	3.024390000	-2.406391000	-4.914663000
6	4.806297000	-0.645905000	-7.833245000
1	5.094181000	0.985955000	-6.475444000
6	4.276535000	-1.911875000	-8.018234000
1	3.219422000	-3.530059000	-7.093333000
1	5.312929000	-0.145630000	-8.648044000
1	4.369784000	-2.409090000	-8.974097000
6	5.075708000	0.685016000	-3.779254000
6	6.311768000	0.050642000	-3.762636000
6	4.972671000	1.984845000	-3.297754000
6	7.425809000	0.709376000	-3.273936000
1	6.395754000	-0.959523000	-4.139826000
6	6.086633000	2.632432000	-2.795175000
1	4.014020000	2.485299000	-3.319072000
6	7.318873000	1.999957000	-2.782997000
1	8.382577000	0.204294000	-3.267664000
1	5.992748000	3.644181000	-2.423252000
1	8.189985000	2.510256000	-2.395156000

PTZ4

6	-3.043419000	-3.612295000	-0.766555000
6	-3.330431000	-2.458892000	-0.027765000
6	-2.631465000	-1.289407000	-0.231371000
6	-1.642924000	-1.197250000	-1.220113000
6	-1.346269000	-2.345484000	-1.951659000
6	-2.028502000	-3.518552000	-1.723767000
6	-1.547484000	1.253858000	-1.175034000
6	-2.512571000	1.395259000	-0.170067000
6	-3.095172000	2.616359000	0.093110000
1	-3.835532000	2.687979000	0.872533000
6	-2.716296000	3.766252000	-0.608966000
6	-1.743274000	3.614380000	-1.601505000
6	-1.172470000	2.394506000	-1.882313000
1	-4.091897000	-2.490864000	0.733719000
1	-0.573861000	-2.323629000	-2.703342000
1	-1.772882000	-4.389108000	-2.313853000
1	-1.428228000	4.477382000	-2.173557000
1	-0.426117000	2.330174000	-2.657552000
7	-0.970893000	0.007652000	-1.459583000
16	-2.910721000	0.043287000	0.881910000
6	0.215785000	-0.011040000	-2.265772000
6	1.448857000	0.002424000	-1.637964000
6	0.150026000	-0.016357000	-3.650652000
6	2.611519000	0.014006000	-2.386511000
1	1.492739000	-0.002527000	-0.557161000
6	1.309955000	0.009244000	-4.401933000
1	-0.814362000	-0.018817000	-4.141583000
6	2.549804000	0.027824000	-3.773490000
1	3.574969000	0.007553000	-1.896461000
1	1.259799000	0.034124000	-5.481616000
6	-3.686095000	-4.906055000	-0.665400000
6	-3.223703000	5.112761000	-0.439441000
1	-2.816370000	5.776702000	-1.194494000
1	-3.292577000	-5.592022000	-1.408037000
6	-4.645527000	-5.484940000	0.096924000
6	-4.058159000	5.763104000	0.406917000
6	-4.955238000	-6.847289000	-0.229235000
6	-5.404514000	-4.901222000	1.225158000
7	-5.174968000	-7.931967000	-0.527286000
8	-6.300468000	-5.770521000	1.710714000
8	-5.269680000	-3.800592000	1.690949000
1	-6.755747000	-5.333645000	2.445290000
6	-4.776763000	5.226483000	1.583520000
6	-4.248214000	7.159711000	0.138825000
7	-4.369944000	8.272304000	-0.108052000
8	-4.746981000	4.095112000	1.989677000
8	-5.496848000	6.178587000	2.190698000
1	-5.935787000	5.767012000	2.949419000
7	3.733773000	0.073005000	-4.535038000
6	4.743438000	1.024063000	-4.407039000
6	4.818940000	2.124423000	-3.564257000
6	5.754418000	0.744361000	-5.339417000
6	5.936620000	2.931392000	-3.658808000
1	4.032200000	2.353140000	-2.859614000
6	6.869178000	1.569669000	-5.414196000
6	6.956883000	2.657880000	-4.569563000
1	6.019431000	3.794775000	-3.012087000
1	7.656929000	1.363463000	-6.127078000
1	7.819737000	3.308141000	-4.615078000
6	4.093899000	-0.815959000	-5.544380000
6	5.339976000	-0.433405000	-6.065547000
6	3.430629000	-1.943830000	-6.007446000
6	5.920243000	-1.180948000	-7.082022000
6	4.028830000	-2.672399000	-7.017684000
1	2.481623000	-2.251908000	-5.592245000
6	5.259387000	-2.296260000	-7.555904000
1	6.879485000	-0.894895000	-7.493670000
1	3.530951000	-3.555230000	-7.396245000
1	5.698511000	-2.887932000	-8.347421000

PTZ5

6	1.102343000	3.747015000	-0.121739000
6	1.854258000	2.628310000	0.257048000
6	1.266587000	1.392800000	0.415989000

6	-0.097044000	1.195635000	0.153409000
6	-0.851424000	2.309711000	-0.214077000
6	-0.264327000	3.546911000	-0.341284000
6	0.046666000	-1.251619000	0.144755000
6	1.423085000	-1.291910000	0.408034000
6	2.149220000	-2.450266000	0.240664000
1	3.209763000	-2.437916000	0.430381000
6	1.530215000	-3.646230000	-0.142636000
6	0.149423000	-3.603960000	-0.360765000
6	-0.574675000	-2.442290000	-0.232227000
1	2.907553000	2.738650000	0.455281000
1	-1.908168000	2.205169000	-0.398650000
1	-0.883482000	4.386353000	-0.630298000
1	-0.369988000	-4.508305000	-0.650443000
1	-1.635867000	-2.457947000	-0.420125000
7	-0.689193000	-0.067379000	0.259451000
16	2.245181000	0.100840000	1.097463000
6	-2.120737000	-0.159179000	0.184484000
6	-2.861598000	-0.261485000	1.348502000
6	-2.782932000	-0.168127000	-1.032920000
6	-4.236698000	-0.369499000	1.308036000
1	-2.350670000	-0.256343000	2.302630000
6	-4.156408000	-0.274239000	-1.091829000
1	-2.213593000	-0.096738000	-1.951196000
6	-4.922629000	-0.377994000	0.082524000
1	-4.784180000	-0.438258000	2.235896000
1	-4.640160000	-0.289623000	-2.056869000
6	1.571944000	5.100753000	-0.319849000
6	2.149234000	-4.938689000	-0.336026000
1	1.409025000	-5.684089000	-0.606937000
1	0.759790000	5.748703000	-0.632083000
6	2.746313000	5.773544000	-0.231230000
6	3.396317000	-5.468290000	-0.275077000
6	2.686573000	7.167397000	-0.565167000
6	4.073390000	5.260246000	0.170013000
7	2.592872000	8.276006000	-0.841136000
8	5.017021000	6.205962000	0.059944000
8	4.329765000	4.150824000	0.557651000
1	5.853465000	5.808710000	0.342990000
6	4.669730000	-4.790751000	0.046992000
6	3.487378000	-6.869347000	-0.569844000
7	3.515096000	-7.988655000	-0.815861000
8	4.819430000	-3.630284000	0.325942000
8	5.702543000	-5.643426000	-0.005889000
1	6.500041000	-5.139719000	0.212815000
7	-6.278841000	-0.484562000	0.032433000
6	-7.049992000	-0.406460000	-1.197019000
6	-8.489289000	-0.288207000	-0.709743000
6	-7.113257000	-0.704584000	1.202086000
6	-8.475096000	-1.052094000	0.611158000
1	-8.533338000	-2.125560000	0.423254000
1	-9.296400000	-0.784456000	1.273136000
1	-9.205629000	-0.682109000	-1.428175000
1	-8.734312000	0.760537000	-0.533410000
1	-6.908146000	-1.303562000	-1.811935000
1	-6.748605000	0.454588000	-1.797153000
1	-6.717237000	-1.511758000	1.821816000
1	-7.165831000	0.195506000	1.826493000

PTZ6

6	3.890678000	1.494935000	-0.403569000
6	2.750957000	0.789787000	-0.007352000
6	1.570234000	1.442174000	0.272115000
6	1.450009000	2.827991000	0.116325000
6	2.587754000	3.537474000	-0.263446000
6	3.774995000	2.883073000	-0.509686000
6	-0.998420000	2.813972000	0.128952000
6	-1.101549000	1.427164000	0.287478000
6	-2.277910000	0.761393000	0.021570000
1	-2.311703000	-0.309525000	0.137495000
6	-3.429122000	1.453104000	-0.364762000
6	-3.329673000	2.842136000	-0.475221000
6	-2.147569000	3.510112000	-0.241114000
1	2.797512000	-0.281105000	0.104996000
1	2.546457000	4.610055000	-0.367747000
1	4.636650000	3.467399000	-0.806623000
1	-4.200236000	3.416182000	-0.766201000
1	-2.119058000	4.582846000	-0.347948000
7	0.223245000	3.471846000	0.339875000
16	0.243376000	0.510464000	0.951365000
6	5.183666000	0.924221000	-0.731491000
6	-4.717999000	0.867092000	-0.681965000
1	-5.424434000	1.622915000	-1.015081000
1	5.881054000	1.689572000	-1.061895000
6	5.712500000	-0.313122000	-0.728572000
6	5.144484000	-1.613821000	-0.331087000
6	5.774000000	-3.964959000	-0.093812000
1	6.338583000	-4.668285000	-0.701701000
6	6.093080000	-4.202399000	1.365227000
8	6.512314000	-3.385230000	2.128316000
8	5.826398000	-5.471685000	1.699785000
1	6.035346000	-5.577567000	2.639721000
7	6.095709000	-2.621576000	-0.493891000
16	8.600367000	-3.260959000	-1.205987000
16	7.380825000	-0.557684000	-1.248153000
8	4.035491000	-1.859296000	0.077015000
6	-5.234055000	-0.375545000	-0.668025000
6	-4.651896000	-1.666907000	-0.260572000
6	-6.837684000	-2.346868000	-0.872684000
7	-5.592895000	-2.685736000	-0.413651000
6	7.335997000	-2.266089000	-0.952500000
6	-5.257491000	-4.022557000	-0.002735000
1	-4.189601000	-4.174190000	-0.148905000
6	-5.578633000	-4.252548000	1.457090000
8	-6.003353000	-3.432468000	2.213996000
8	-5.307697000	-5.518635000	1.799970000
8	-3.539910000	-1.897185000	0.148285000
1	-5.518184000	-5.619688000	2.740107000
16	-8.091176000	-3.357596000	-1.117670000
16	-6.900871000	-0.641447000	-1.182003000
6	0.215482000	4.901683000	0.455317000
6	0.204711000	5.715144000	-0.668268000
6	0.218596000	5.464916000	1.719771000
6	0.196694000	7.091477000	-0.522343000
1	0.202526000	5.265742000	-1.652782000
6	0.210667000	6.842727000	1.863028000
6	0.199632000	7.656579000	0.743269000
1	0.188160000	7.724505000	-1.399587000
1	0.193310000	8.732521000	0.855501000
1	0.213050000	7.279643000	2.852566000
1	0.227124000	4.815704000	2.584992000
1	4.708419000	-4.127741000	-0.244692000
1	-5.811934000	-4.736790000	-0.607197000

PTZ7

6	3.960048000	-0.083650000	-0.815211000
6	2.780788000	-0.674068000	-0.352062000
6	1.598870000	0.033351000	-0.296626000
6	1.522912000	1.356917000	-0.746093000
6	2.696656000	1.948282000	-1.210118000
6	3.879940000	1.244400000	-1.238765000
6	-0.937321000	1.443548000	-0.718658000
6	-1.096012000	0.124282000	-0.276520000
6	-2.323316000	-0.502232000	-0.321451000

1	-2.401700000	-1.527011000	0.004956000
6	-3.468670000	0.175514000	-0.750465000
6	-3.308476000	1.505292000	-1.144944000
6	-2.077695000	2.122444000	-1.144038000
1	2.797371000	-1.693310000	-0.001321000
1	2.687244000	2.972309000	-1.547201000
1	4.769363000	1.741393000	-1.604547000
1	-4.171590000	2.071304000	-1.471027000
1	-2.004870000	3.147943000	-1.468579000
7	0.316494000	2.071560000	-0.717969000
16	0.229696000	-0.735411000	0.493706000
6	5.264247000	-0.713078000	-0.906849000
6	-4.818324000	-0.352874000	-0.809957000
1	-5.524368000	0.410883000	-1.124999000
1	5.982063000	-0.062909000	-1.399943000
6	5.789108000	-1.890062000	-0.519970000
6	5.199315000	-3.016951000	0.223450000
6	5.829591000	-5.147340000	1.246749000
1	6.437938000	-5.994148000	0.937317000
6	6.055905000	-4.882186000	2.718294000
8	6.416083000	-3.845813000	3.189605000
8	5.782068000	-5.977734000	3.438757000
1	5.932002000	-5.761176000	4.370954000
7	6.165554000	-3.998521000	0.449356000
16	8.714216000	-4.767601000	0.124739000
16	7.484702000	-2.250451000	-0.848566000
8	4.064877000	-3.151246000	0.613057000
6	-5.397023000	-1.543860000	-0.572227000
6	-4.824948000	-2.836079000	-0.156385000
6	-7.117795000	-3.407613000	-0.332883000
7	-5.831965000	-3.798681000	-0.069089000
6	7.430675000	-3.776056000	-0.025590000
6	-5.503760000	-5.126796000	0.374705000
1	-4.484685000	-5.353964000	0.066399000
6	-5.584069000	-5.246269000	1.879715000
8	-5.802790000	-4.349605000	2.637190000
8	-5.358831000	-6.510885000	2.260194000
8	-3.672539000	-3.108646000	0.076163000
1	-5.406537000	-6.539864000	3.227248000
16	-8.450512000	-4.342321000	-0.268586000
16	-7.140147000	-1.729889000	-0.770346000
6	0.358933000	3.477811000	-0.995317000
6	0.371225000	3.920257000	-2.307955000
6	0.376207000	4.386190000	0.064427000
6	0.401727000	5.268439000	-2.598587000
1	0.354863000	3.189481000	-3.106175000
6	0.404044000	5.771468000	-0.233043000
6	0.362493000	3.975314000	1.439108000
6	0.417518000	6.213270000	-1.575729000
1	0.410956000	5.599874000	-3.628937000
6	0.414940000	6.723653000	0.823383000
6	0.373331000	4.881699000	2.433571000
1	0.343135000	2.918325000	1.663046000
6	0.444219000	7.621623000	-1.842180000
6	0.439777000	8.106685000	0.530807000
6	0.398714000	6.289773000	2.167917000
1	0.362614000	4.555111000	3.465913000
6	0.454753000	8.520735000	-0.841310000
1	0.454760000	7.947504000	-2.874583000
6	0.447580000	9.021221000	1.580344000
6	0.407240000	7.237465000	3.186798000
1	0.473952000	9.581620000	-1.057235000
6	0.431240000	8.588965000	2.893573000
1	0.466124000	10.080490000	1.357213000
1	0.394526000	6.904930000	4.217059000
1	0.437115000	9.312723000	3.697659000
1	4.777237000	-5.381078000	1.096320000
1	-6.188099000	-5.840275000	-0.078482000

PTZ8

6	0.214639000	3.942708000	-0.614066000
6	1.047939000	2.884953000	-0.235621000
6	0.539924000	1.627667000	0.015732000
6	-0.823737000	1.350632000	-0.145053000
6	-1.656171000	2.402351000	-0.524389000
6	-1.147004000	3.662041000	-0.745726000
6	-0.556361000	-1.089467000	-0.030874000
6	0.833944000	-1.050598000	0.137064000
6	1.603666000	-2.185659000	-0.010573000
1	2.673182000	-2.114715000	0.109734000
6	1.020198000	-3.427582000	-0.283364000
6	-0.369796000	-3.461559000	-0.410300000
6	-1.139945000	-2.327144000	-0.296399000
1	2.104994000	3.058331000	-0.111355000
1	-2.714563000	2.234649000	-0.642178000
1	-1.827332000	4.452579000	-1.035666000
1	-0.862440000	-4.403784000	-0.613576000
1	-2.208804000	-2.405494000	-0.409567000
7	-1.341670000	0.066940000	0.073647000
16	1.634698000	0.419756000	0.676851000
6	0.614322000	5.310976000	-0.883824000
6	1.703198000	-4.695848000	-0.449890000
1	0.996742000	-5.493759000	-0.662860000
1	-0.233053000	5.909694000	-1.207384000
6	1.763132000	6.008834000	-0.822006000
6	3.120596000	5.609958000	-0.414206000
6	5.352079000	6.552028000	-0.076277000
1	5.976095000	7.258614000	-0.618900000
6	5.508604000	6.790240000	1.408492000
8	4.622405000	7.029108000	2.172203000
8	6.796099000	6.685304000	1.763959000
1	6.850133000	6.835670000	2.719391000
7	3.984855000	6.703214000	-0.495774000
16	4.272352000	9.310905000	-1.057685000
16	1.777502000	7.720295000	-1.251372000
8	3.512498000	4.524955000	-0.059388000
6	2.979360000	-5.118400000	-0.407163000
6	4.233640000	-4.392910000	-0.147687000
6	5.054606000	-6.592701000	-0.471796000
7	5.315621000	-5.272635000	-0.216535000
6	3.465656000	7.903983000	-0.901385000
6	6.640511000	-4.790970000	0.067259000
1	6.691121000	-3.737799000	-0.204003000
6	6.975032000	-4.924382000	1.535767000
8	6.224042000	-5.294017000	2.387247000
8	8.242006000	-4.553182000	1.762816000
8	4.388638000	-3.218686000	0.084480000
1	8.408483000	-4.642591000	2.712923000
16	6.148376000	-7.795568000	-0.576079000
16	3.349695000	-6.820385000	-0.686320000
6	-2.766760000	-0.094800000	0.091413000
6	-3.489184000	-0.240074000	-1.082678000
6	-3.435486000	-0.127000000	1.301844000
6	-4.857121000	-0.417608000	-1.048291000
1	-2.974404000	-0.207226000	-2.034388000
6	-4.803868000	-0.316726000	1.345239000
6	-5.538519000	-0.467697000	0.169582000
1	-5.406967000	-0.522510000	-1.972460000
1	-5.308392000	-0.355699000	2.299899000
1	-2.873319000	-0.021597000	2.220315000
1	5.675771000	5.536613000	-0.297737000
1	7.365694000	-5.352209000	-0.517446000
7	-6.924574000	-0.672674000	0.203262000
6	-7.721668000	-0.086460000	1.217664000

6	-8.695869000	-0.838978000	1.863803000
6	-7.552335000	1.248222000	1.570312000
6	-9.485861000	-0.264129000	2.843047000
1	-8.833032000	-1.876770000	1.592352000
6	-8.334772000	1.812376000	2.561288000
1	-6.803273000	1.842197000	1.064599000
6	-9.307612000	1.061831000	3.200884000
1	-10.239802000	-0.862851000	3.336992000
1	-8.191468000	2.851992000	2.825076000
1	-9.922645000	1.507454000	3.970787000
6	-7.548873000	-1.485127000	-0.777548000
6	-8.695427000	-1.044291000	-1.428015000
6	-7.026941000	-2.734542000	-1.093741000
6	-9.305542000	-1.840822000	-2.380658000
1	-9.106472000	-0.074722000	-1.181536000
6	-7.632218000	-3.519411000	-2.058231000
1	-6.142606000	-3.086042000	-0.579616000
6	-8.775323000	-3.078631000	-2.704903000
1	-10.196599000	-1.484836000	-2.880800000
1	-7.214566000	-4.489092000	-2.295531000
1	-9.250358000	-3.696565000	-3.454671000

PTZ9

6	0.388516000	3.596880000	-1.611738000
6	1.161160000	2.662867000	-0.916441000
6	0.602591000	1.508634000	-0.411349000
6	-0.748167000	1.206813000	-0.614595000
6	-1.520197000	2.130023000	-1.316499000
6	-0.962617000	3.295099000	-1.795696000
6	-0.537766000	-1.144069000	0.057833000
6	0.836756000	-1.066205000	0.311278000
6	1.605547000	-2.203176000	0.440110000
1	2.664188000	-2.103465000	0.618419000
6	1.032045000	-3.476619000	0.375195000
6	-0.346279000	-3.542980000	0.159966000
6	-1.114190000	-2.410883000	-0.001864000
1	2.206958000	2.858050000	-0.743119000
1	-2.568960000	1.941711000	-1.482952000
1	-1.595369000	3.991071000	-2.331443000
1	-0.831340000	-4.509382000	0.108494000
1	-2.174023000	-2.516041000	-0.170593000
7	-1.304773000	0.017692000	-0.118038000
16	1.601571000	0.488071000	0.613364000
6	0.851425000	4.856824000	-2.164056000
6	1.723441000	-4.744754000	0.515918000
1	1.036473000	-5.580331000	0.410751000
1	0.067185000	5.351103000	-2.731312000
6	2.001743000	5.553509000	-2.126188000
6	3.280662000	5.283295000	-1.444044000
6	5.457131000	6.315575000	-1.019565000
1	6.167474000	6.877131000	-1.621982000
6	5.402161000	6.911037000	0.369107000
8	4.416388000	7.296172000	0.921913000
8	6.626135000	6.938835000	0.912696000
1	6.544914000	7.315132000	1.801608000
7	4.167761000	6.338884000	-1.656741000
16	4.584916000	8.739437000	-2.777721000
16	2.118019000	7.104899000	-2.957921000
8	3.596599000	4.321309000	-0.787437000
6	2.992386000	-5.126920000	0.747603000
6	4.222804000	-4.342674000	0.956543000
6	5.066647000	-6.547194000	1.166341000
7	5.304358000	-5.198299000	1.168774000
6	3.737638000	7.397759000	-2.411296000
6	6.600030000	-4.644067000	1.455377000
1	6.683911000	-3.681369000	0.954151000
6	6.799819000	-4.428813000	2.938606000
8	5.977498000	-4.608778000	3.785497000
8	8.040887000	-3.986424000	3.180875000
8	4.362402000	-3.143914000	0.947591000
1	8.123521000	-3.851792000	4.136602000
16	6.164133000	-7.727102000	1.403010000
16	3.386418000	-6.842993000	0.855223000
6	-2.731883000	-0.087335000	-0.055016000
6	-3.478004000	-0.507221000	-1.145579000
6	-3.374246000	0.247443000	1.124577000
6	-4.853639000	-0.604378000	-1.052401000
1	-2.976193000	-0.773253000	-2.066722000
6	-4.751820000	0.170818000	1.215512000
6	-5.500618000	-0.261212000	0.128968000
1	-5.433067000	-0.957657000	-1.893807000
1	-5.253544000	0.456872000	2.129200000
1	-2.786925000	0.584467000	1.968114000
1	5.787798000	5.281614000	-0.937525000
1	7.371435000	-5.315102000	1.084604000
7	-6.903364000	-0.351165000	0.223799000
6	-7.610637000	-1.024225000	1.217179000
6	-8.985819000	-0.874101000	0.980051000
6	-7.138323000	-1.780946000	2.280764000
6	-9.902065000	-1.470414000	1.836836000
6	-8.069022000	-2.366579000	3.117301000
1	-6.080385000	-1.918484000	2.451718000
6	-9.438682000	-2.210648000	2.905456000
1	-10.965066000	-1.358763000	1.666691000
1	-7.725126000	-2.960167000	3.953962000
1	-10.140025000	-2.679195000	3.582262000
6	-7.819782000	0.227344000	-0.650724000
6	-9.119629000	-0.074498000	-0.215026000
6	-7.597718000	1.020309000	-1.768408000
6	-10.212976000	0.407141000	-0.923207000
6	-8.701147000	1.488756000	-2.455442000
1	-6.598801000	1.274192000	-2.092411000
6	-9.998167000	1.183125000	-2.044169000
1	-11.220007000	0.180004000	-0.598074000
1	-8.553267000	2.108194000	-3.330137000
1	-10.839888000	1.563812000	-2.606312000

PTZ10

6	3.769549000	0.253770000	-0.688936000
6	2.618629000	-0.387071000	-0.221500000
6	1.466427000	0.321058000	0.044954000
6	1.394949000	1.702481000	-0.171620000
6	2.542934000	2.345833000	-0.630956000
6	3.694276000	1.634989000	-0.884503000
6	-1.044932000	1.806767000	-0.070181000
6	-1.207982000	0.430190000	0.134185000
6	-2.424665000	-0.185330000	-0.067481000
1	-2.506417000	-1.250084000	0.080897000
6	-3.556257000	0.551606000	-0.432126000
6	-3.394951000	1.931536000	-0.581692000
6	-2.175530000	2.547367000	-0.411042000
1	2.633941000	-1.449508000	-0.040273000
1	2.532138000	3.411782000	-0.793297000
1	4.562278000	2.167301000	-1.252465000
1	-4.248809000	2.539156000	-0.853579000
1	-2.098289000	3.614565000	-0.544134000
7	0.210122000	2.414332000	0.064911000
16	0.115853000	-0.528222000	0.785422000
6	5.029992000	-0.378201000	-1.030032000
6	-4.884300000	0.029455000	-0.689381000
1	-5.562731000	0.819880000	-0.999843000
1	5.697286000	0.316977000	-1.532796000
6	5.556674000	-1.606627000	-0.876295000
6	5.037997000	-2.801951000	-0.190117000

6	5.695727000	-5.088002000	0.389899000
1	6.143119000	-5.904001000	-0.173121000
6	6.236995000	-5.103508000	1.801559000
8	6.757632000	-4.184501000	2.358302000
8	6.038666000	-6.303581000	2.363299000
1	6.388467000	-6.266210000	3.265774000
7	5.968598000	-3.840006000	-0.270048000
16	8.372021000	-4.649227000	-1.136864000
16	7.161326000	-1.966568000	-1.514790000
8	3.984232000	-2.943429000	0.380351000
6	-5.467717000	-1.182913000	-0.649838000
6	-4.940994000	-2.504355000	-0.269747000
6	-7.175274000	-3.066210000	-0.825724000
7	-5.937144000	-3.473601000	-0.407320000
6	7.148278000	-3.595558000	-0.920217000
6	-5.658283000	-4.835315000	-0.039397000
1	-4.593077000	-5.016590000	-0.171800000
6	-6.013489000	-5.109738000	1.404452000
8	-6.400861000	-4.301514000	2.193237000
8	-5.818629000	-6.403607000	1.693448000
8	-3.830741000	-2.792836000	0.105357000
1	-6.047904000	-6.533013000	2.625632000
16	-8.483082000	-4.011347000	-1.050595000
16	-7.164196000	-1.355172000	-1.103606000
6	0.281623000	3.845794000	0.129949000
6	0.232336000	4.636945000	-1.007481000
6	0.418310000	4.461922000	1.361944000
6	0.311497000	6.011630000	-0.921268000
1	0.126466000	4.169637000	-1.978550000
6	0.504639000	5.835091000	1.466282000
6	0.452160000	6.648796000	0.323374000
1	0.256174000	6.598159000	-1.826189000
1	0.623580000	6.279971000	2.442751000
1	0.464511000	3.850309000	2.253645000
1	4.616668000	-5.225314000	0.434109000
1	-6.223979000	-5.508925000	-0.679190000
6	0.590453000	8.722565000	1.680151000
6	0.607647000	8.891660000	-0.731589000
6	0.402821000	10.179979000	1.274275000
1	1.553396000	8.568717000	2.182567000
1	-0.192659000	8.382072000	2.360779000
6	1.005518000	10.233590000	-0.126666000
1	1.342328000	8.536336000	-1.457374000
1	-0.359092000	8.956043000	-1.245916000
1	-0.661318000	10.419013000	1.234303000
1	0.873223000	10.869941000	1.972304000
1	0.651632000	11.077329000	-0.716121000
1	2.092922000	10.302345000	-0.063876000
7	0.535031000	8.005771000	0.417465000
